# Lipidomics and biodistribution of extracellular vesicles‐secreted by hepatocytes from Zucker lean and fatty rats

**DOI:** 10.1002/jex2.140

**Published:** 2024-02-22

**Authors:** Maria Azparren‐Angulo, Justyna Mleczko, Oihane E. Alboniga, Sergei Kruglik, Jean‐Michel Guigner, Esperanza Gonzalez, Clara Garcia‐Vallicrosa, Jordi Llop, Cristina Simó, Cristina Alonso, Marta Iruarrizaga, Felix Royo, Juan M. Falcon‐Perez

**Affiliations:** ^1^ Exosomes Laboratory, Center for Cooperative Research in Biosciences (CIC bioGUNE) Basque Research and Technology Alliance (BRTA), Derio Bizkaia Spain; ^2^ Metabolomics Platform, CICbioGUNE‐BRTA, CIBERehd Bizkaia Technology Park, Derio Bizkaia Spain; ^3^ Laboratoire Jean Perrin Sorbonne Université, CNRS UMR 8237, 4 place Jussieu Paris France; ^4^ L'Institut de Minéralogie, de Physique des Matériaux et de Cosmochimie Sorbonne Université, CNRS, IRD, MNHN Paris France; ^5^ CIC biomaGUNE Basque Research and Technology Alliance (BRTA), Paseo Miramón 182, San Sebastian Guipúzcoa Spain; ^6^ OWL Metabolomics. Derio Bizkaia Spain; ^7^ Centro de Investigación Biomédica en Red de enfermedades hepáticas y digestivas (CIBERehd) Instituto de Salud Carlos III Madrid Spain; ^8^ IKERBASQUE Basque Foundation for Science, Bilbao Bizkaia Spain

**Keywords:** biodistribution, EVs, lipidomics, liver, metabolic syndrome, Raman microspectroscopy, RTM

## Abstract

Extracellular vesicles (EVs) have been involved in metabolic syndrome, although their specific role in the development of the pathology is still unknown. To further study the role of EVs, we have analysed by Raman tweezers microspectroscopy and mass spectrometry‐based lipidomics the small EVs population secreted by fatty (ZF) and lean (ZL) hepatocytes obtained from Zucker rats. We have also explored in vivo and ex vivo biodistribution of these EVs through fluorine‐18‐radiolabelling using a positron emission tomography imaging. Based on the proportion of proteins to lipids and the types of lipids, our results indicate that within the range of small EVs, primary hepatocytes secrete different subpopulations of particles. These differences were observed in the enrichment of triglyceride species in EVs secreted by ZF hepatocytes. Biodistribution experiments showed accumulation in the brain, heart, lungs, kidney and specially in bladder after intravenous administration. In summary, we show that EVs released by a fatty hepatocytes carry a different lipid signature compared to their lean counterpart. Biodistribution experiment has shown no difference in the distribution of EVs secreted by ZF and ZL hepatocytes but has given us a first view of possible target organs for these particles. Our results might open a door to both pathology studies and therapeutic interventions.

## 1 INTRODUCTION

Metabolic syndrome is a group of conditions associated with an increased risk of coronary heart disease, stroke, certain cancers, sleep apnea, liver and gall bladder disease, osteoarthritis, and gynaecological problems (Guh et al., 2009; Williams et al., 2015). Clinically diagnosing metabolic syndrome requires the presence of at least three of the following symptoms; central obesity, hypertension, dyslipidemia, hyperglycemia, and/or insulin resistance (Ford, 2004; Srikanthan et al., 2016). Due to socio‐economic and cultural reasons, obesity has reached pandemic proportions in recent decades (Villalobos, 2016). In order to stop the progression of damage induced by metabolic alterations, it is necessary to develop tools for properly evaluating the severity of the condition and monitoring recovery following therapeutic intervention. As metabolic syndrome results from the intricate interplay of different organs involved in systemic metabolism, the study of circulating messengers able to determine the crosstalk between organs is a key element, and in that sense, extracellular vesicles (EVs) have been appointed as one of the best candidates for biomarker discovery (Martínez & Andriantsitohaina, 2017).

EVs are heterogeneous membrane‐enclosed vesicles released by cells (Vallabhaneni et al., 2015). Based on their size and mechanism of formation, EVs can be divided into three major types: exosomes (30‐250 nm), formed from multivesicular bodies of the endocytic and secretory pathways; microvesicles (50‐5000 nm), originated by the outward budding of the plasma membrane; and apoptotic bodies (<1‐5 μm), formed from cells undergoing apoptosis (Abels & Breakefield, 2016; Kalra et al., 2012; Théry et al., 2009). Thanks to their circulating nature, EVs transport various signals encoded by proteins, nucleic acids (mRNA and miRNA), lipids and metabolites (Vlassov et al., 2012). These signals are carried from donor cells to recipient cells, allowing cell‐to‐cell communication, either in a paracrine or endocrine manner (Bianco et al., 2005; Peña‐Altamira et al., 2018). Recipient cells will be influenced by EVs cargo inducing changes in gene or protein expressions, proliferation, differentiation or cellular metabolism (Vlassov et al., 2012).

Numerous reports support the involvement of EVs in the development of metabolic syndrome and fat‐associated liver diseases, with a growing interest in the potential of circulating EVs as biomarkers (detailed review can be found in Azparren‐Angulo et al., 2019). Murine models of obesity proved the increased level of EVs released in pathological condition, and the presence of circulating EVs containing perilipin A, a protein present in tissue‐derived EVs (Camino et al., 2020; Eguchi et al., 2017). In addition, there are already well‐established protocols to characterize the lipidomic profile of human serum EVs, facilitating the detection of possible lipid biomarker candidates for different diseases (Chen et al., 2019; Sun et al., 2019). The study of lipids in circulating EVs has already been applied to early prediction of preterm birth (Gao et al., 2020), and prostate cancer classification (Brzozowski et al., 2018).

Recent research has explored protein differences in EVs secreted by primary hepatocytes from Zucker rats, a well‐established model of metabolic syndrome, and their metabolic impact on adipocytes (Mleczko et al., 2022). Following on from this research line, our focus remains on investigating Zucker rats as a target for further investigation on the role of EVs in metabolic syndrome development.

Based on the last published paper detailing differences in proteomic composition between vesicles from ZL and ZF rats, this study hypothesised that the composition, biodistribution, and impact of EVs on recipient organs differ in an obesity model. Therefore, this study aims to molecularly characterize these EVs and examine their biodistribution in pathologies associated with obesity.

## 2 MATERIAL AND METHODS

### 2.1 Animal procedure

All animal experimentation was conducted in accordance with the Spanish Guide for the Care and Use of Laboratory Animals (RD 53/2013 — BOE‐A‐2013‐1337) and regional Basque Country ethical committee (P‐CBG‐CBBA‐0219). Male Zucker rats were obtained from Charles River Laboratories (France) at 10–12 weeks of age, both fatty (ZF) and its lean (ZL) control, (ZUC(Orl)‐*Lepr* ^fa^ strain) and they were used as a source of primary hepatocytes. A total of 38 rats were used in the different experiments, 19 ZL and 19 ZF, always conducting the experiments in parallel with one ZF and its respective ZL pair. Additionally, 14‐week‐old male Wistar rats weighing between 300 and 400 g were also acquired from Charles River Laboratories (France). All animals were maintained with the same conditions and diet, in an environmentally controlled room at 22°C on a 12 h light/ dark cycle and provided with standard diet, 14% protein and 3.7% fat die, (Rodent Maintenance Diet, Harlan Teklad Global Diet 2014) and water *ad libitum*. Surgery was performed under anaesthetic inhalation of isoflurane (IsoFLO, Abbott Laboratories) in pure oxygen as the carrier gas (5% for induction, 2–3% for maintenance), and all efforts were made to minimize the animal suffering. For liver perfusion, a two‐step collagenase technique was employed which involves sequential perfusion of the liver with ethylenediaminetetraacetic acid and collagenase (Siendones et al., 2005). Briefly, a laparotomy was performed to expose the liver, portal vein, and infrahepatic inferior vena cava. The portal vein was cannulated to ensure continuous perfusion at a rate of 6.3 mL/min with 150 mL of Leffert buffer (Leffert buffer: 4‐(2‐hydroxyethyl)−1‐piperazineethanesulfonic acid (HEPES) 10 mM, KCl 3 mM, NaCl 130 mM, NaH2PO4‐H2O mM, glucose 10 mM) with 250 mM ethylene glycol bis(β‐aminoethyl ether)‐N,N,N',N'‐tetraacetic acid (EGTA). To enable flow, the infrahepatic inferior vena cava was opened. Subsequently, at the same rate, 150 mL of Leffert buffer was perfused, followed by 150 mL of Leffert buffer perfused with 30,000 units of collagenase type I (Worthington Biochemical Corp, Lakewood, NJ) and 110 mM CaCl2 at a serial rate change. Following the removal of catheters, the liver was manually sliced and explanted into a Petri dish containing Dulbecco's Modified Eagle Medium (DMEM). Finally, the DMEM containing the liver cells was filtered through a cell strainer and divided into two 50 mL Falcon tubes.

### 2.2 Primary rat hepatocyte culture, EV production and isolation

One rat liver was used for each liver primary culture. Hepatocytes obtained from the perfused liver were disaggregated and centrifuged at 200 × g for 4 min at 4°C to separate the cells and eliminate perfusion debris. The resulting cell pellet was then resuspended in 30 mL of cold DMEM and 15 mL of 90% Percoll for further purification through Percoll density separation (90% Percoll in Phosphate‐Buffered Saline [PBS]), achieved by centrifugation at 200 × g for 10 min. The resulting cell pellet was washed three times with 30 mL of cold DMEM and centrifuged at 200 × g for 5 min, and the final pellet was resuspended in 50 mL DMEM for cell counting and subsequent seeding.

Afterwards, hepatocytes (>99% purity) were seeded in collagen‐coated 150 mm dishes, at 10 million cells per dish and cultured in DMEM at 37°C and 5% of CO_2_, until cells were completely attached. To avoid EVs contamination from FBS, DMEM medium containing 10% FBS was ultracentrifuged for 16 h at 100,000x g generating an EV‐depleted medium. Once cells were completely attached after 4 h, medium was replaced by EV‐depleted 25‐mM HEPES (4‐[2‐hydroxyethyl]‐1‐piperazine ethanesulfonic acid) DMEM medium containing 10% FBS for 36 h. For EV isolation, conditioned medium from 240 × 10^6^ cells (300 mL of culture medium) was centrifuged at 2,000 × g for 10 min to remove dead cells and cellular debris. Resulting supernatant was ultracentrifuged at 10,000 × g for 30 min to remove large EVs and obtained pellet was discarded. The obtained supernatant was further ultracentrifuged at 100,000 × g for 90 min, and the resulting pellet containing the small EVs was resuspended in PBS and ultracentrifuged again at 100,000 × g for 90 min. Final pellet was resuspended in 150 μL of PBS, aliquoted and stored at −80°C.

### 2.3 Western blot analysis

Samples were prepared in 4 × LDS sample buffer (NuPAGE, life technologies), then boiled (5 min, 37°C; 10 min, 65°C; 15 min 90°C) and finally centrifugated (13,000 × rpm, 1 min) before being applied to the gel. For electrophoresis a 4–12% Bis‐Tris Precast gels (NuPAGE, Invitrogen) and MOPS SDS running buffer (NuPAGE, Life Technologies) were used and samples were run for 90 min at 120 V. Proteins were transferred into polyvinylidene fluoride membranes (PVDF, Immobilon‐P) using NuPAGE transfer buffer (Life Technologies). Then, membranes were blocked with 5% non‐fat dry milk in TPBS (5% tween in PBS), 1 h at room temperature. Once the membranes were blocked, an overnight incubation with primary antibodies in 5% BSA TPBS was performed at 4°C. Membranes were washed three times with TPBS and secondary antibody was incubated in 5% non‐fat dry milk in TPBS for 45 min at room temperature. Membranes were then washed three times with TPBS. The membranes were visualised using the ImageQuant LAS 4000 with Clarity™ Western Enhanced Chemiluminescence Blotting Substrate (BioRad). The complete list of primary antibodies can be found in Table S1.

### 2.4 Cryo‐electron microscopy

Cryo‐electron microscopy was used for imaging of EVs samples, which was carried out as previously described (Royo et al., 2013). EVs preparations were directly adsorbed onto glow‐discharged holey carbon grids (QUANTIFOIL). The grids were blotted at 95% humidity and rapidly immersed in liquid ethane with the aid of VITROBOT (Maastricht Instruments BV). Images of the vitrified samples were acquired at liquid nitrogen temperature using a JEM‐2200FS/CR transmission cryo‐electron microscope (JEOL) operated at an accelerating voltage of 200 kV and equipped with a field emission gun.

### 2.5 Bodipy staining analysis

Percoll‐purified primary hepatocytes from Zucker rats were seeded in collagen‐coated six‐well dishes containing a coverslip at a density 5 × 10^5^ cells per well and cultured in DMEM at 37°C and 5% of CO_2_. Once cells were completely attached, the coverslips were fixed for 30 min in formaldehyde (PBS 4% formaldehyde). After fixation, the cells were incubated with BODIPY^R^493/503 (4,4‐Difluoro‐1,3,5,7,8‐Pentamethyl‐4‐Bora‐3a,4a‐Diaza‐s‐Indacene) (Thermo‐Fisher Scientific) at a concentration of 10 μg/mL for 45 min at room temperature, protected from light. Coverslips were then washed twice with PBS for 10 min, and subsequently mounted onto glass slides in Fluoromont G (Southern Biotechnology Associates) containing 0.7 g/mL 4′,6‐diamidino‐2‐phenylindole (DAPI) to stain the nucleus. Bodipy solution was pre‐clarified by centrifugation at 10,000 × g for 15 min. Images were acquired with a Confocal Microscope using a Leica SP8 confocal Microscope (Leica Microsystems Inc.).

### 2.6 Flow cytometry analysis

Flow cytometry analysis was performed using the BD FACSCantoTM II system (BD Biosciences). Data was analysed using the BD FACSDivaTM software (BD Biosciences) and later processed using FlowJo 7.6 software. Flow cytometry analysis of Bodipy staining was employed to quantify lipid accumulation. In brief, cells were lifted with TrypLE TM (Gibco) and fixed in suspension with 2% paraformaldehyde solution for 10 min and subsequently washed three times, 10 min each, with D‐PBS. Then, cells were stained with Bodipy (2 μg/mL) for 30 min at room temperature, protected from light. Finally, cells were washed twice with PBS and resuspended in D‐PBS (400 μL) for flow cytometry analysis.

### 2.7 Lipase assay

Triglyceride content of Zucker rat hepatocytes and their secreted EVs was measured using lipase digestion assay. In brief, cell pellets were lysed with ice‐cold lysis buffer (50 mM Tris pH 7.5, 150 mM NaCl, 1% Triton X‐100, 1X protease inhibitor) for 15 min on ice following 15 min centrifugation at 10,000 × g. The resulting cellular extract was transferred to a fresh tube, and the amount of protein was determined by Bradford assay. In the case of EVs, the same protocol was followed although the lysis buffer was 2X concentrated to avoid sample dilution. All samples in the assay were done in duplicate and adjusted to the same volume (30 μL) and to equal protein concentration. Samples were incubated at 700 × g for 5 min. Subsequently, 7.5 μL of the Lipoprotein Lipase (10 mg/mL, dissolved in dH_2_0) was added to each tube following overnight incubation at 37°C. Afterwards, the assay was developed with 200 μL/sample of the Free Glycerol Reagent (FGR) and absorbance was read on 96‐well plates at 540 nm wavelength with Spectramax M3 spectrophotometer (Molecular Devices, LLC). Absorbance values were normalized to the protein concentration.

### 2.8 Raman tweezers microspectroscopy analysis

Three preparation of EVs obtained from ZF and ZL rats were used for Raman Tweezers Microspectroscopy Analysis. Raman spectra were recorded using a home‐built Raman Tweezers microspectroscope (RTM) setup described elsewhere (Kruglik et al., 2019; Tatischeff et al., 2012), with a few modifications. In brief, near‐infrared excitation at 780 nm (180 mW at sample position) was provided by continuous‐wave Ti:Sapphire laser (Spectra‐Physics model 3900S) pumped by 1.8 W of green light from Verdi DPSS laser (Coherent). Raman scattering was excited using long working distance water‐immersion objective (Olympus LUMFL, M = 60X, NA = 1.1) directly plunged into the droplet (∼100 μL) of water buffer solution containing bioparticles. Our Raman microscope works in an upright configuration, thus avoiding problems with sedimentation of particles or debris from sample solution and providing minimal optical path through various optical components, hence reducing the background Raman signal. The focal point, which creates an optical trap, was located inside the sample droplet, about 2 mm above the stage slide made of CaF_2_. Raman scattering light was collected in a back‐scattering geometry, filtered off with a matched pair of dichroic beam‐splitter and long‐pass sharp‐edge filter (Semrock), and focused into the 50‐μm × 10‐mm spectrometer entrance slit by a 75 mm achromatic focusing lens. Spectrometer with a 500 mm focal length (Acton SpectraPro 2500i) and a 400 mm‐1 grating optimized for 850 nm was used for spectral dispersion. Raman signal was registered at the exit spectrometer port by a back‐illuminated CCD detector (Princeton Instruments SPEC‐10 400BR/LN), cooled to 140 K using liquid nitrogen. The effective volume of Raman signal collection within the optical trap was estimated as a cylinder with a diameter of ∼0.86 μm and axial height of ∼3.5 μm, giving the volume of ∼2 μm^3^.

Spectral resolution of our RTM setup was about 5 cm^−1^. Frequency calibration was performed using Raman lines of toluene with absolute accuracy ± 2 cm^−1^ and relative frequency position accuracy ± 1 cm^−1^. Raman spectra were acquired using WinSpec software; further data treatment was performed using IgorPro for Windows software. The details of Raman spectra acquisition and subsequent data treatment were described elsewhere (Kruglik et al., 2019; Tatischeff et al., 2012).

### 2.9 Biodistribution experiments

Fluorine‐18 was produced in a Cyclone 18/9 cyclotron (IBA) by proton irradiation of an ^18^O‐enriched water target via the ^18^O(p,n)^18^F nuclear reaction. 6‐[^18^F]fluoronicotinic acid 2,3,5,6‐tetrafluorophenyl ester ([^18^F]F‐PyTFP) was synthesised using a TRACERlab FX_FN_ synthesis module (GE Healthcare), following a previously described procedure (Hortelao et al., 2021). In brief, aqueous [^18^F]F^−^ was first trapped in an ion‐exchange cartridge (Sep‐Pak® Accell Plus QMA Light) and subsequently eluted to the reactor vessel with a solution of Kryptofix K_2.2.2_/K_2_CO_3_ in a mixture of water and acetonitrile. After azeotropic drying of the solvent, a solution of the precursor (10 mg) in a mixture of *tert*‐butanol/acetonitrile (4/1) was added and heated at 40°C for 15 min. The reaction mixture was then diluted with 1 mL of acetonitrile and 1 mL of water, and purified by high performance liquid chromatography (HPLC) using a Nucleosil 100–7 C18 column (Machery‐Nagel, Düren, Germany) as the stationary phase and 0.1% aqueous trifluoroacetic acid (TFA)/acetonitrile (25/75) as the mobile phase at a flow rate of 3 mL/min. The desired fraction (retention time: 22–23 min) containing [^18^F]F‐PyTFP) was collected, diluted with water (30 mL), and reformulated using a C18 cartridge (Sep‐Pak® Light, Waters). Radiochemical purity was always >95% as determined by radio‐HPLC, using a Mediterranean C18 column (4.6 × 150 mm, 5 μm) as stationary phase and water/acetonitrile (both containing 0.1% TFA) as the mobile phase, using the following gradient: 0–1 min 25% acetonitrile; 9–12 min 90% acetonitrile; 13–15 min 25% acetonitrile; flow rate: 1.5 mL/min).

Radiolabelling of EVs (ZL and ZF) was performed by mixing 3 μL of 10 mM PBS pH 7.4, 3 μL of [^18^F]F‐PyTFP in acetonitrile, and 6 μL of EVs solution (∼ 6 μg/μL). The samples for injection were prepared from 36 ug of protein, that are approximately 1.9 E10 vesicles measured by NTA. This protein quantification was done previous to radioactivity label, and to ensure an equal amount of EVs injected we confirm that radiolabel efficiency is equal for both preparations. The reaction mixture was heated at 40°C for 15 min, diluted with 100 μL of PBS pH 7.4, and purified by size exclusion chromatography using Illustra™ Nap™−5 Sephadex™ columns G‐25 DNA grade (GE Healthcare), preconditioned with PBS (10 mL, 10 mM pH7.4). The fractions containing pure labelled EVs were collected, the amount of radioactivity was measured in a dose calibrator, and quality control was performed by radio‐thin layer chromatography (radio‐TLC), using iTLC‐SG chromatography paper (Agilent Technologies) and dichloromethane/methanol (2/1) as stationary and mobile phases, respectively.

Positron emission tomography (PET) scans (10 min duration) were performed in anaesthetized (5% isoflurane in pure oxygen for induction; 2% isoflurane in pure oxygen for maintenance) wild‐type male rats at ca. 1, 3 and 6 h after intravenous injection of labelled EVs using a small animal PET scanner (β Cube, MOLECUBES, Gent, Belgium). Wild‐type Wistar rats were selected as the injection group to ensure that lean vesicles were not preferentially favoured when injected into lean rats, thus equalizing the variability between the two groups effectively. After each PET scan, computerised tomography (CT) images were acquired using a small animal scanner (X Cube, MOLECUBES, Gent, Belgium) to provide anatomical information as well as the attenuation map for the later image reconstruction. PET images were reconstructed with OSEM‐3D iterative algorithm. Images were analysed using π‐MOD image analysis software (π‐MOD Technologies Ltd, Zurich, Switzerland). Volumes of interest (VOIs) were manually drawn in major organs, namely brain, lungs, kidneys, liver, bladder and heart, using the CT images for anatomical reference. VOIs were then transferred to PET images and the concentration of radioactivity in each organ was determined at all time points, normalised to the injected activity, and expressed as percentage of injected dose per cm^3^ of tissue (%ID/cm^3^).

After the imaging sessions, animals were sacrificed by exsanguination followed by perfusion with saline solution, organs of interest were collected and weighed, and the radioactivity was measured in a gamma counter (Wallach Wizard, PerkinElmer, Waltham). The uptake was calculated as a percentage of the injected dose per gram of tissue (% ID/g).

### 2.10 Ultra‐high performance liquid chromatography coupled to mass spectrometry (UHPLC‐MS)‐based lipidomic analysis

UHPLC‐MS‐based lipidomic analysis was performed by OWL metabolomics. Lipid extraction was accomplished by fractionating the cells and EVs into pools of species with similar physicochemical properties, using appropriate combinations of organic solvents (modified protocol from Barr et al. (2012, 2010). Cellular pellets of primary hepatocytes obtained from four ZF and four ZL rats and their secreted EVs were resuspended in cold water and vortexed. Primary hepatocytes were obtained directly after perfusion (T0 hepatocytes) and collected EVs were released by hepatocytes during the time frame of 4 h to 36 h. Proteins were precipitated from the lysed cell samples by adding methanol and after a brief vortex, chloroform was added to samples. Both extraction solvents were spiked with lipids not detected in unspiked cell extracts (internal standards: SM (d18:1/16:0), PE (17:0/17:0), PC (19:0/19:0), TG (13:0/13:0/13:0), Cer (d18:1/17:0), and ChoE (12:0)). Samples were incubated at −20 ˚C for 30 min and after vortexing them, 500 μL were collected. Cells and EVs extracts were mixed with ammonium hydroxide in water (pH 9), vortexed and incubated for 1 h at −20 ˚C. After centrifugation at 18,000x g for 15 min at 4°C, the organic phase was collected and dried under vacuum. Dried extracts were then reconstituted in acetonitrile/isopropanol (1/1), resuspended with agitation for 10 min, centrifuged at 18,000x g for 5 min at 4°C, and transferred to plates for UHPLC‐MS analysis. Finally, quality control (QC) sample was prepared as a pool of samples in order to assess data quality and reproducibility (Van Der Kloet et al., 2009).

Chromatographic separation and mass spectrometry detection conditions are summarized in Supplementary Table S2. The overall quality of the analysis procedure was monitored using the QC samples as well as the lock mass accuracy injected simultaneously in the LC‐MS system. Retention time stability throughout the course of the run is less than 6 s variation (injection‐to‐injection) and mass accuracy less than 5 ppm for m/z 400–1000, and 1.2 mDa for m/z 50–400. Randomized sample injections were performed, with the QC sample interspersed throughout the entire batch run. QC samples were used not only to assess data quality but also to correct any analytical or response factor differences throughout the analysis (Van Der Kloet et al., 2009).

All data were processed using the TargetLynx application manager for MassLynx 4.1 software (Waters Corp., Milford). A set of predefined retention time, mass‐to‐charge ratio pairs, RT‐*m/z*, corresponding to lipids included in the analysis are fed into the program. Associated extracted ion chromatograms (mass tolerance window = 0.05 Da) were then peak‐detected and noise‐reduced in both the LC and MS domains such that only true lipid related features are processed by the software. A list of chromatographic peak areas was then generated for each sample injection. Considering the analytical variations as well as biological variation, normalization procedure was performed as follows. First, each lipid intensity was divided by the most appropriate internal standard in each sample, and then QC response was corrected to remove any analytical variation following the procedure described by Martínez‐Arranz et al. (2015). Finally, biological variability associated to EVs was corrected using the total amount of protein in each sample to normalized them.

Multivariate principal component analysis (PCA) and orthogonal partial least squares discriminant analysis (OPLS‐DA) modelling were performed with the software SIMCA 14.1 (Umetrics, Malmo). Model quality was assessed using R2 and Q2 values, which indicate the explained fraction of variance and the goodness of prediction, respectively. The Q2 parameter was calculated by sevenfold cross‐validation and CV‐ANOVA was used as model validation criteria.

Univariate statistical analyses were also performed, calculating unpaired fold‐changes, unpaired Student's t‐test *p*‐value (or Welch´s t test where unequal variances were found) and log2 (fold‐change) for comparisons between cells or EVs obtained from ZF rats and cells or EVs obtained from ZL rats. In this last case, also the corrected *q*‐value (False Discovery Rate which was the *p*‐value corrected by Benjamini and Hochberg approach) was calculated and included in Table 1. The selection criteria for significant metabolites wad q‐value ≤ 0.05.

**TABLE 1:** **Analysis of 199 lipids** found in cells or EVs obtained from ZL rats compared to cells or EVs obtained from ZF rats (n=4 per condition). List of metabolites classification, tentative annotation and results obtained in the analysis The colour code represents the fold change, while the degree of grey correlates with the significance of the regulation. Unpaired Student's t‐test p‐values and Benjamini‐Hochberg correction q‐values are also shown.

			**Cells: ZL Vs ZF**			**EVs: ZL Vs ZF**		
**Metabolic class**		**Individual notation**	**log2** **(fold change)**	**Student's t‐test (p)**	**q‐value**	**log2** **(fold change)**	**Student's t‐test (p)**	**q‐value**
**Glycerolipids**	**DAG**	*DG(30:0)*	**2.62**	2.52E‐01	0.46	‐0.05	8.30E‐01	0.91
		*DG(30:1)*	**3.15**	1.86E‐01	0.43	‐0.08	9.10E‐01	0.93
		*DG(32:0)*	**2.39**	1.29E‐01	0.40	0.14	4.89E‐01	0.64
		*DG(32:1)*	**1.56**	2.17E‐01	0.45	0.23	5.33E‐01	0.68
		*DG(32:2)*	**2.13**	2.30E‐01	0.45	‐0.02	9.79E‐01	0.98
		*DG(34:1)*	**1.27**	2.31E‐01	0.45	0.63	5.22E‐02	0.32
		*DG(34:2)*	**0.69**	4.49E‐01	0.65	0.11	8.14E‐01	0.89
		*DG(36:1)*	**1.30**	1.26E‐01	0.40	0.33	3.39E‐01	0.53
		*DG(36:2)*	**0.79**	3.76E‐01	0.57	0.58	3.25E‐01	0.53
		*DG(36:3)*	**‐0.24**	7.99E‐01	0.88	0.17	7.93E‐01	0.89
		*DG(38:5)*	**‐0.61**	5.53E‐01	0.73	‐0.27	4.59E‐01	0.63
		*DG(38:6)*	**0.68**	5.54E‐01	0.73	0.04	9.21E‐01	0.94
	**TAG**	*TG(42:0)*	**4.23**	8.37E‐02	0.38	0.07	8.81E‐01	0.93
		*TG(42:1)*	**1.51**	3.22E‐02	0.25	0.39	4.07E‐01	0.58
		*TG(43:0)*	**1.08**	2.24E‐01	0.45	‐0.22	6.92E‐01	0.81
		*TG(44:0)*	**3.22**	1.54E‐01	0.40	0.79	3.37E‐01	0.53
		*TG(44:1)*	**3.69**	4.70E‐02	0.31	0.45	4.74E‐01	0.63
		*TG(44:2)*	**1.58**	9.88E‐04	0.18	0.08	8.67E‐01	0.93
		*TG(45:0)*	**1.29**	1.79E‐01	0.42	‐0.14	7.00E‐01	0.81
		*TG(45:1)*	**1.77**	1.11E‐01	0.39	‐0.53	3.30E‐01	0.53
		*TG(46:0)*	**2.56**	2.50E‐01	0.46	0.87	2.79E‐01	0.49
		*TG(46:1)*	**3.39**	1.01E‐01	0.38	0.87	2.90E‐01	0.51
		*TG(46:2)*	**2.92**	8.52E‐03	0.25	0.25	6.49E‐01	0.78
		*TG(46:3)*	**1.13**	1.37E‐02	0.25	0.05	9.33E‐01	0.95
		*TG(47:0)*	**1.06**	3.29E‐01	0.53	‐0.29	3.97E‐01	0.58
		*TG(47:1)*	**2.09**	3.32E‐02	0.25	‐0.26	5.36E‐01	0.68
		*TG(47:2)*	**1.29**	1.41E‐02	0.25	‐0.59	2.74E‐01	0.49
		*TG(48:0)*	**1.64**	3.39E‐01	0.54	0.96	9.30E‐02	0.33
		*TG(48:1)*	**2.89**	1.34E‐01	0.40	0.93	1.25E‐01	0.33
		*TG(48:2)*	**2.72**	9.35E‐02	0.38	0.76	2.62E‐01	0.48
		*TG(48:3)*	**2.01**	1.43E‐02	0.25	0.07	8.83E‐01	0.93
		*TG(48:4)*	**0.18**	7.18E‐01	0.84	‐0.82	1.21E‐01	0.33
		*TG(49:0)*	**2.78**	1.99E‐01	0.45	0.11	6.86E‐01	0.81
		*TG(49:1)*	**2.65**	9.24E‐02	0.38	0.19	5.17E‐01	0.67
		*TG(49:2)*	**1.96**	4.62E‐02	0.31	‐0.19	5.66E‐01	0.70
		*TG(49:3)*	**0.62**	2.70E‐01	0.47	‐0.80	8.38E‐02	0.33
		*TG(50:0)*	**3.17**	2.57E‐01	0.46	1.71	1.16E‐01	0.33
		*TG(50:1)*	**2.16**	1.40E‐01	0.40	1.13	1.90E‐02	0.31
		*TG(50:2)*	**1.69**	1.56E‐01	0.40	0.93	4.94E‐02	0.32
		*TG(50:3)*	**1.60**	8.00E‐02	0.38	0.51	3.32E‐01	0.53
		*TG(51:1)*	**3.42**	7.11E‐02	0.37	0.54	8.60E‐02	0.33
		*TG(51:2)*	**2.07**	5.28E‐02	0.31	0.48	1.11E‐01	0.33
		*TG(51:3)*	**0.40**	4.94E‐01	0.68	‐0.15	5.55E‐01	0.70
		*TG(52:0)*	**3.83**	1.69E‐01	0.42	1.38	1.51E‐01	0.35
		*TG(52:1)*	**3.99**	8.66E‐02	0.38	1.86	2.80E‐02	0.31
		*TG(52:2)*	**1.28**	2.17E‐01	0.45	1.24	6.63E‐03	0.31
		*TG(52:3)*	**0.52**	4.82E‐01	0.67	0.75	7.12E‐02	0.32
		*TG(52:4)*	**0.87**	2.78E‐01	0.47	0.41	4.26E‐01	0.59
		*TG(52:5)*	**1.00**	2.01E‐01	0.45	0.59	2.98E‐01	0.51
		*TG(52:4)*	**0.47**	4.31E‐01	0.63	0.69	2.21E‐01	0.44
		*TG(53:0)*	**0.03**	9.77E‐01	0.99	‐0.07	8.66E‐01	0.93
		*TG(53:1)*	**3.06**	6.74E‐02	0.37	0.33	3.90E‐01	0.57
		*TG(53:2)*	**2.30**	1.11E‐01	0.39	0.57	1.05E‐01	0.33
		*TG(53:3)*	**0.87**	2.87E‐01	0.48	0.35	3.48E‐01	0.54
		*TG(53:4)*	**‐1.06**	8.95E‐02	0.38	‐0.21	6.18E‐01	0.75
		*TG(54:0)*	**0.93**	2.73E‐01	0.47	0.58	3.68E‐01	0.55
		*TG(54:1)*	**4.25**	8.83E‐02	0.38	1.47	7.97E‐02	0.32
		*TG(54:2)*	**2.25**	1.50E‐01	0.40	1.96	1.20E‐02	0.31
		*TG(54:3)*	**0.25**	7.31E‐01	0.84	0.94	2.54E‐02	0.31
		*TG(54:4)*	**‐0.09**	8.91E‐01	0.93	0.79	1.84E‐01	0.40
		*TG(54:5)*	**‐1.03**	1.76E‐01	0.42	0.51	1.52E‐01	0.35
		*TG(54:5)*	**0.98**	1.64E‐01	0.41	1.19	1.42E‐01	0.35
		*TG(54:6)*	**‐1.20**	1.50E‐01	0.40	0.38	4.14E‐01	0.58
		*TG(54:6)*	**0.21**	7.71E‐01	0.87	‐0.13	8.85E‐01	0.93
		*TG(54:7)*	**0.59**	5.45E‐01	0.73	0.22	6.58E‐01	0.79
		*TG(55:1)*	**1.70**	2.82E‐02	0.25	‐0.64	3.76E‐01	0.55
		*TG(55:2)*	**1.00**	4.77E‐02	0.31	‐0.06	8.63E‐01	0.93
		*TG(55:3)*	**‐0.46**	2.54E‐01	0.46	‐0.16	6.96E‐01	0.81
		*TG(55:4)*	**‐2.15**	2.27E‐03	0.18	‐0.06	9.36E‐01	0.95
		*TG(56:0)*	**‐0.57**	4.15E‐01	0.62	‐0.15	7.82E‐01	0.88
		*TG(56:1)*	**3.26**	1.42E‐01	0.40	‐0.05	9.10E‐01	0.93
		*TG(56:2)*	**1.71**	1.53E‐01	0.40	0.82	6.70E‐02	0.32
		*TG(56:3)*	**‐0.37**	5.70E‐01	0.74	1.04	3.82E‐02	0.32
		*TG(56:5)*	**0.79**	2.25E‐01	0.45	1.06	1.27E‐01	0.33
		*TG(56:6)*	**0.09**	8.91E‐01	0.93	0.99	1.46E‐01	0.35
		*TG(56:7)*	**0.33**	6.24E‐01	0.78	1.05	1.79E‐01	0.40
		*TG(56:8)*	**‐0.01**	9.84E‐01	0.99	‐0.08	8.63E‐01	0.93
		*TG(58:1)*	**1.02**	1.26E‐01	0.40	‐0.25	6.82E‐01	0.81
		*TG(58:2)*	**0.56**	2.31E‐01	0.45	‐0.49	2.71E‐01	0.49
		*TG(58:3)*	**0.01**	9.81E‐01	0.99	0.24	5.62E‐01	0.70
		*TG(58:5)*	**‐0.24**	5.10E‐01	0.70	0.95	1.14E‐01	0.33
		*TG(58:6)*	**‐0.67**	2.04E‐01	0.45	0.87	2.81E‐01	0.49
		*TG(58:8)*	**‐1.47**	2.68E‐01	0.47	0.33	4.48E‐01	0.61
		*TG(58:9)*	**‐0.50**	4.79E‐01	0.67	0.36	5.27E‐01	0.68
**Sterols**	**Cho**	*Cholesterol*	**0.41**	4.27E‐01	0.63	‐0.28	4.03E‐01	0.58
	**ChoE**	*ChoE(18:2)*	**‐0.29**	7.16E‐01	0.84	0.05	8.97E‐01	0.93
		*ChoE(20:4)*	**0.85**	2.25E‐01	0.45	0.21	3.70E‐01	0.55
		*ChoE(20:5)*	**0.20**	6.51E‐01	0.80	0.05	8.08E‐01	0.89
		*ChoE(22:6)*	**1.37**	2.09E‐01	0.45	0.11	6.87E‐01	0.81
**Glycerophospholipids**	PE	*PE(16:0/16:0)*	**0.51**	5.70E‐01	0.74	‐0.96	1.18E‐01	0.33
		*PE(16:0/16:1)*	**‐0.05**	9.41E‐01	0.97	‐1.93	4.13E‐02	0.32
		*PE(16:0/18:1)*	**‐0.65**	5.74E‐01	0.74	‐1.30	1.14E‐01	0.33
		*PE(16:0/18:2)*	**‐1.62**	2.33E‐01	0.45	‐2.16	5.99E‐02	0.32
		*PE(16:0/20:4)*	**‐0.28**	7.96E‐01	0.88	‐2.12	6.07E‐02	0.32
		*PE(18:0/18:1)*	**‐0.81**	4.44E‐01	0.65	‐0.74	2.25E‐01	0.44
		*PE(16:0/22:6)*	**‐0.07**	9.51E‐01	0.97	‐1.55	2.93E‐02	0.31
		*PE(18:0/20:4)*	**‐0.14**	8.90E‐01	0.93	‐1.42	6.90E‐02	0.32
		*PE(20:4/18:2)*	**‐1.81**	2.14E‐01	0.45	‐1.15	9.90E‐02	0.33
		*PE(38:5)*	**‐1.16**	3.74E‐01	0.57	‐1.85	4.91E‐02	0.32
		*PE(18:0/22:6)*	**0.27**	8.08E‐01	0.88	‐0.89	4.56E‐02	0.32
		*PE(18:0/22:4)*	**0.30**	6.76E‐01	0.81	‐0.75	2.39E‐01	0.46
		*PE(18:1e/22:4)*	**‐0.82**	1.72E‐01	0.42	‐0.78	1.37E‐01	0.34
		*PE(O‐16:0/18:1)*	**‐1.18**	5.60E‐02	0.31	‐0.68	2.48E‐01	0.46
		*PE(P‐16:0/18:2)*	**‐1.95**	2.92E‐01	0.49	‐0.72	4.71E‐01	0.63
		*PE(P‐16:0/20:4)*	**‐0.43**	6.99E‐01	0.83	‐0.56	5.00E‐01	0.66
		*PE(P‐18:0/18:1)*	**‐0.56**	4.64E‐01	0.66	‐0.87	3.12E‐01	0.52
		*PE(P‐16:0/22:6)*	**‐0.25**			‐0.62	4.75E‐01	0.63
		*PE(P‐18:0/20:4)*	**‐1.31**	3.26E‐01	0.53	‐0.67	3.65E‐01	0.55
		*PE(P‐18:1/20:4)*	**‐0.72**	5.82E‐01	0.74	‐0.45	6.44E‐01	0.78
		*PE(P‐18:0/22:5) + PE(P‐20:1/20:4)*	**1.28**	3.74E‐01	0.57	‐0.71	2.97E‐01	0.51
	PC	*PC(30:0)*	**‐0.29**	7.71E‐01	0.87	‐1.36	1.21E‐01	0.33
		*PC(14:0/16:1)*	**‐1.70**	2.34E‐02	0.25	‐1.24	6.91E‐02	0.32
		*PC(14:0/18:2)*	**‐2.20**	3.00E‐02	0.25	‐1.51	3.18E‐02	0.31
		*PC(16:0/16:0)*	**‐0.33**	7.34E‐01	0.84	‐0.62	3.07E‐01	0.51
		*PC(32:1)*	**‐0.11**	9.18E‐01	0.95	‐1.28	6.16E‐02	0.32
		*PC(15:0/18:2)*	**‐3.14**	1.13E‐01	0.39	‐1.54	1.00E‐01	0.33
		*PC(16:0/17:0)*	**‐0.21**	8.44E‐01	0.90	‐0.96	1.50E‐01	0.35
		*PC(33:1)*	**‐1.81**	1.40E‐01	0.40	‐0.88	1.25E‐01	0.33
		*PC(14:0/20:4)*	**‐1.47**	1.48E‐01	0.40	‐0.80	6.04E‐02	0.32
		*PC(16:0/18:0)*	**‐0.22**	7.33E‐01	0.84	‐0.53	4.21E‐01	0.59
		*PC(16:0/18:1)*	**‐0.58**	5.88E‐01	0.74	‐0.50	1.82E‐01	0.40
		*PC(16:0/18:2)*	**‐0.61**	5.57E‐01	0.73	‐1.80	6.79E‐02	0.32
		*PC(15:0/20:4)*	**‐2.22**	1.03E‐01	0.38	‐1.44	3.05E‐02	0.31
		*PC(17:0/18:1)*	**‐1.64**	9.22E‐03	0.25	‐0.40	2.49E‐01	0.46
		*PC(17:1/18:1)*	**‐2.59**	2.45E‐02	0.25	‐0.89	1.17E‐01	0.33
		*PC(16:0/20:4)*	**‐0.10**	9.20E‐01	0.95	‐0.90	5.79E‐02	0.32
		*PC(16:0/20:5)*	**1.12**	2.78E‐01	0.47	‐0.64	2.08E‐01	0.43
		*PC(16:1/20:4)*	**‐1.85**	1.06E‐01	0.38	‐1.18	1.05E‐02	0.31
		*PC(18:0/18:1)*	**0.13**	8.41E‐01	0.90	‐0.24	5.96E‐01	0.73
		*PC(18:0/18:2)*	**‐0.41**	6.55E‐01	0.80	‐1.11	7.24E‐02	0.32
		*PC(18:2/18:2)*	**‐1.73**	1.15E‐01	0.39	‐0.87	6.29E‐02	0.32
		*PC(36:3)*	**1.39**	1.33E‐01	0.40	‐1.09	7.78E‐02	0.32
		*PC(15:0/22:6)*	**‐2.27**	1.58E‐02	0.25	‐1.12	1.25E‐01	0.33
		*PC(17:0/20:4)*	**‐1.45**	2.27E‐02	0.25	‐1.21	1.16E‐02	0.31
		*PC(37:2)*	**‐2.01**	3.37E‐02	0.25	‐0.64	1.13E‐01	0.33
		*PC(16:0/22:6)*	**‐0.14**	8.85E‐01	0.93	‐0.61	3.07E‐01	0.51
		*PC(18:0/20:3)*	**0.15**	8.44E‐01	0.90	0.04	9.07E‐01	0.93
		*PC(18:0/20:4)*	**0.01**	9.88E‐01	0.99	‐0.12	5.93E‐01	0.73
		*PC(18:2/20:4)*	**‐1.00**	2.55E‐01	0.46	‐1.15	1.38E‐02	0.31
		*PC(20:0/18:2)*	**‐0.64**	2.06E‐01	0.45	‐0.40	2.13E‐01	0.43
		*PC(18:0/22:4)*	**0.22**	6.65E‐01	0.81	0.26	3.51E‐01	0.54
		*PC(18:0/22:5)*	**‐1.55**	1.49E‐01	0.40	‐0.07	8.07E‐01	0.89
		*PC(18:0/22:6)*	**0.21**	7.88E‐01	0.87	‐0.15	7.61E‐01	0.87
		*PC(18:1/22:6)*	**‐1.89**	1.58E‐01	0.40	‐1.23	2.47E‐02	0.31
		*PC(20:0/20:4)*	**‐3.86**	9.62E‐03	0.25	‐1.25	6.41E‐03	0.31
		*PC(40:5)*	**0.58**	4.58E‐01	0.66	0.66	7.90E‐02	0.32
		*PC(40:8)*	**‐1.04**	1.31E‐01	0.40	‐0.55	1.08E‐01	0.33
		*PC(40:0)*	**‐0.31**	7.20E‐01	0.84	0.16	7.59E‐01	0.87
		*PC(38:5)*	**‐1.02**	4.01E‐01	0.60	‐1.38	1.75E‐02	0.31
		*PC(38:5)*	**0.23**	7.73E‐01	0.87	‐0.06	9.09E‐01	0.93
		*PC(O‐16:0/16:0)*	**‐0.98**	9.19E‐02	0.38	‐0.47	3.05E‐01	0.51
		*PC(O‐22:1/20:4)*	**‐0.90**	1.40E‐02	0.25	‐0.25	3.60E‐01	0.55
		*PC(O‐34:0)*	**‐1.66**	2.10E‐02	0.25	‐0.57	3.28E‐01	0.53
		*PC(O‐34:1)*	**‐0.99**	3.14E‐01	0.52	‐0.49	3.24E‐01	0.53
		*PC(O‐38:5)*	**‐0.94**	3.56E‐01	0.55	‐0.78	2.46E‐01	0.46
		*PC(O‐40:5)*	**‐0.91**	1.73E‐01	0.42	0.01	9.61E‐01	0.97
		*PC(P‐16:0/16:0)*	**‐0.98**	3.37E‐01	0.54	‐0.33	3.99E‐01	0.58
		*PC(P‐18:0/20:4)*	**‐1.70**	5.27E‐02	0.31	‐1.05	2.04E‐01	0.43
	PI	*PI(18:0/20:3)*	**2.36**	2.14E‐01	0.45	‐0.66	1.68E‐01	0.38
		*PI(18:0/20:4)*	**0.39**	6.91E‐01	0.83	‐0.88	6.30E‐02	0.32
**Sphingolipids**	**Cer**	*Cer(d18:1/16:0)*	**‐0.32**	4.72E‐01	0.66	‐0.65	3.51E‐02	0.32
		*Cer(d18:1/18:0)*	**1.00**	1.85E‐01	0.43	0.07	7.73E‐01	0.88
		*Cer(d18:1/19:0)*	**‐0.43**	3.12E‐01	0.52	‐0.09	8.08E‐01	0.89
		*Cer(d18:1/20:0)*	**0.12**	7.88E‐01	0.87	‐0.53	8.93E‐02	0.33
		*Cer(d18:1/22:0)*	**‐0.68**	9.45E‐02	0.38	‐0.49	8.66E‐02	0.33
		*Cer(40:2)*	**‐1.00**	5.45E‐02	0.31	‐0.98	1.26E‐02	0.31
		*Cer(d18:1/23:0)*	**‐1.30**	2.49E‐02	0.25	‐0.76	1.81E‐02	0.31
		*Cer(d18:1/24:0)*	**‐0.77**	1.03E‐01	0.38	‐0.84	4.36E‐02	0.32
		*Cer(d18:1/24:1)+ Cer(d18:2/24:0)*	**‐0.78**	7.80E‐02	0.38	‐0.77	3.31E‐02	0.31
		*Cer(d43:1)*	**‐1.53**	8.24E‐03	0.25	‐1.12	9.35E‐03	0.31
	**SM**	*SM(43:2)*	**1.22**	2.72E‐01	0.47	‐0.82	6.27E‐02	0.32
		*SM(38:0)*	**‐0.85**			‐0.28	5.54E‐01	0.70
		*SM(42:1)*	**‐0.39**	3.45E‐01	0.54	‐1.40	2.50E‐02	0.31
		*SM(32:1)*	**‐1.35**	8.49E‐02	0.38	‐0.73	4.34E‐02	0.32
		*SM(33:1)*	**‐1.14**	1.28E‐01	0.40	‐0.97	9.44E‐02	0.33
		*SM(d18:0/14:0)*	**‐2.18**			‐0.61	2.49E‐01	0.46
		*SM(d18:0/16:0)*	**‐0.67**	5.15E‐01	0.70	‐1.18	1.54E‐01	0.35
		*SM(d18:0/18:0)*	**‐0.40**	6.75E‐01	0.81	‐0.99	1.37E‐01	0.34
		*SM(d18:0/22:0)*	**‐1.84**	3.10E‐02	0.25	‐0.60	1.88E‐01	0.41
		*SM(d18:1/16:0)*	**‐0.41**	6.21E‐01	0.78	‐1.00	2.02E‐01	0.43
		*SM(d18:1/17:0)*	**0.25**	7.78E‐01	0.87	‐0.60	4.21E‐01	0.59
		*SM(d18:1/18:0)*	**0.75**	3.75E‐01	0.57	‐0.45	4.85E‐01	0.64
		*SM(36:2)*	**0.72**	4.69E‐01	0.66	‐0.78	1.93E‐01	0.41
		*SM(38:1)*	**‐0.36**	5.35E‐01	0.72	‐0.50	3.54E‐01	0.54
		*SM(39:1)*	**‐1.22**	2.75E‐02	0.25	‐0.41	2.22E‐01	0.44
		*SM(d18:1/22:0)*	**‐0.67**	2.33E‐01	0.45	‐0.89	2.10E‐01	0.43
		*SM(d18:1/23:0)*	**‐1.31**	5.11E‐02	0.31	‐1.27	7.96E‐02	0.32
		*SM(d18:1/23:1)*	**‐1.07**	3.99E‐02	0.29	‐0.66	9.98E‐02	0.33
		*SM(42:3)*	**‐2.48**			‐1.83	1.01E‐01	0.33
		*SM(d18:1/24:1) + SM(d18:2/24:0)*	**‐0.75**	2.37E‐01	0.45	‐1.03	1.53E‐01	0.35
		*SM(d18:1/25:0)*	**‐1.63**	2.73E‐03	0.18	‐1.33	8.47E‐03	0.31
		*SM(d18:2/16:0)*	**‐1.32**	9.82E‐02	0.38	‐0.78	7.70E‐02	0.32
		*SM(d18:2/20:0)*	**‐1.52**	1.42E‐01	0.40	‐0.89	2.32E‐01	0.45
		*SM(d18:2/22:0)*	**‐1.28**	2.71E‐02	0.25	‐0.73	1.11E‐01	0.33
	**CMH**	*CMH(d18:1/16:0)*	**1.70**	7.59E‐02	0.38	‐1.33	1.38E‐01	0.34
		*CMH(d18:1/22:0)*	**0.46**	1.66E‐02	0.25	‐0.82	2.99E‐02	0.31
		*CMH(d18:1/24:0)*	**‐0.02**	9.28E‐01	0.96	‐1.00	3.98E‐02	0.32
		*CMH(d18:1/24:1)*	**0.55**	6.33E‐01	0.79	‐0.87	1.00E‐01	0.33
		*CMH(d18:1/25:1)*	**‐0.71**	2.49E‐01	0.46	‐1.61	2.07E‐01	0.43

			≤−4	−4	−3	−2	−1	1	2	3	4	≥4		
			p<0.05				p<0.01			p<0.001				

*Note*: List of metabolites classification, tentative annotation and results obtained in the analysis The colour code represents the fold change, while the degree of grey correlates with the significance of the regulation. Unpaired Student's t‐test p‐values and Benjamini‐Hochberg correction q‐values are also shown.

Finally, lipids were identified also by OWL metabolomics based on an in‐house library build up analysing internal standards by MS/MS experiments in positive and negative ionization modes. Experimental *m/z* and RT of the lipids included in this study were compared with those included in the in‐house library to annotate them. RT shift was also considered by comparing the RTs of spiked internal standards in the samples with those RTs in the library to correctly assign lipid identification.

## 3 RESULTS

### 3.1 Molecular characterization of small EVs secreted by hepatocytes from rat Zucker model

Small EVs were isolated from primary hepatocytes of ZL and ZF rats, and as observed through electron microscopy double‐membrane vesicular structures were detected in both (Figure S1A). The composition of primary hepatocytes and their secreted EVs from ZL and ZF were analysed by Western blotting (Figure S1B), loading the same amount of protein measured by Bradford assay. As it can be seen in Figure S1B we were able to detect EV markers AIP1 and CD63 in the preparations, with higher expression in lean‐derived EVs, as previously described (Mleczko et al., 2022). The presence of the mitochondrial protein (COXIV) was undetectable in the vesicles secreted by lean primary hepatocytes; however, its presence was clearly detected in the small EVs released fatty primary hepatocytes, supporting the difference in molecular composition between lean and fatty hepatocyte‐derived EVs. In addition, the lipid droplet marker perilipin (but not adipophilin) was also detected in the EV preparations, although, due to the intrinsic variability of the primary hepatocytes, we could not establish significant differences among ZL and ZF preparations for these proteins. Finally, we also detected apolipoproteins (ApoB), although no significant differences were detected, suggesting no differences in lipoproteins content of lean and fatty hepatocyte derived‐EVs. Entire electrophoretogram of the Western blot analysis could be found in Figure S2.

**FIGURE 1:**
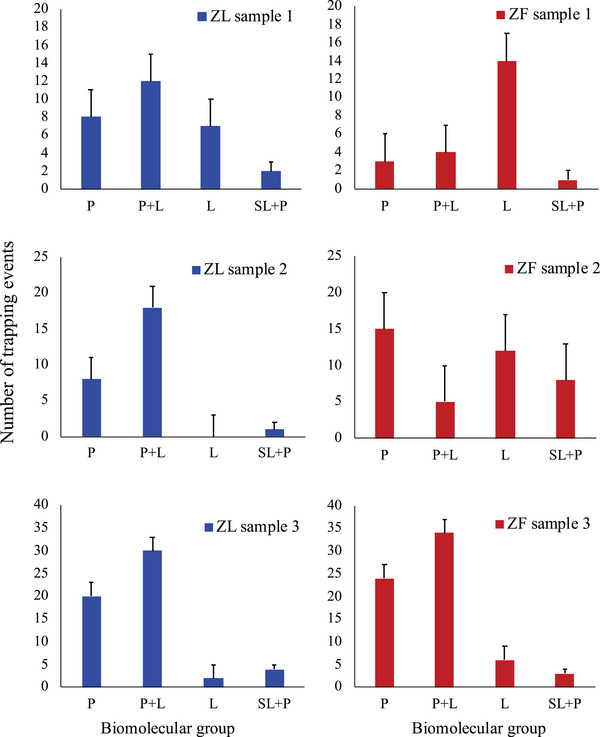
Comparative Raman Tweezers Micrsospectroscopy characterization of EVs from ZL versus ZF hepatocytes, for three different EV preparations. Individual EVs from ZL hepatocytes (left panels, blue bars) were optically trapped and analysed in total 112 times, while EVs from ZF hepatocytes (right panels, red bars) were analysed 129 times. Raman spectra recorded in each trapping event were classified by an educated observer into one of four biomolecular groups: P = dominant proteins; P+L = proteins and lipids in various non‐negligible proportions; L = dominant unsaturated lipids, negligible proteins; SL+P = saturated lipids and proteins in various proportions. Error bars correspond to the uncertainty of spectra attribution to a particular biomolecular group.

Further molecular characterization was performed by RTM (Kruglik et al., 2019) to evaluate the biomolecular composition of individual EVs secreted by ZL and ZF hepatocytes (Figure 1). More than 100 trapping events from each condition were analysed for three independent EV sample preparations; and the spectral profiles of the recorded Raman spectra were found to reflect rather complicated biomolecular composition of the trapped EVs under study. To obtain a semi‐quantitative EVs biomolecular characterization, all the recorded Raman spectra were classified into 4 different groups based on the contribution of proteins and lipids as group L, group P, group P+L, and group SL+P where proteins and saturated lipids contribute in different proportions. Raman spectra of the four different biomolecular groups are deeper explained in Figure S3.

The analysis of RTM characterization of individual EVs presented in Figure 1 allows us to formulate the following qualitative findings. First, for all three ZL samples studied, the group “P+L” corresponds to the core of EVs biomolecular distribution, as expected. In contrast, in the ZF case, only one of three samples (ZF3) shows the same tendency, while for other two ZF samples, the group “P+L” is not dominant, with major contributions being either from lipids (ZF1) or from proteins (ZF2). Second, for all samples studied, the contribution from unsaturated lipids (group “L”) is much stronger for ZF as compared to the corresponding ZL sample; this finding is well beyond the experimental error. Third, both ZL and ZF samples possess marginal amount of EVs with major contribution from saturated lipids (group “SL+P”), the sample ZF2 being the only one exception.

Finally, one important finding consists in the apparent strong heterogeneity of biomolecular distribution in EVs from one sample to another. Considering this finding, it is clear that we cannot obtain a meaningful statistic from the limited data presented in Figure 1. Therefore, we refrain from a more detailed analysis, beyond the above formulated qualitative observations; further RTM study on individual EVs from numerous independent samples is required in this case.

### 3.2 Analysis of lipid content of hepatocytes and hepatocytes derived EVs

**FIGURE 2:**
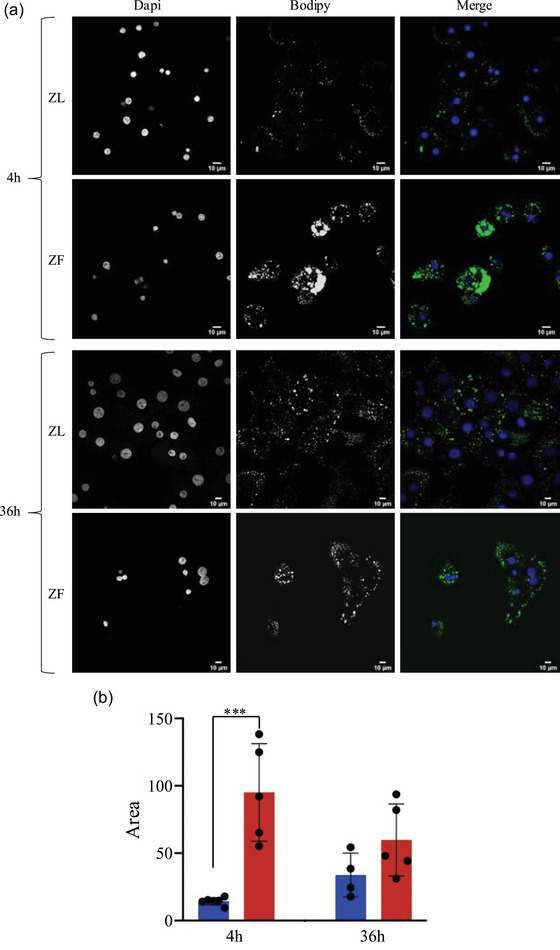
Bodipy staining of lipids from ZL and ZF primary hepatocytes. (a) Confocal representative images were taken 4 h and 36 h after cell seeding. (b) Quantification of the area of Bodipy signal from ZL and ZF hepatocytes 4 h and 36 h after cell seeding. The *p*‐values were denoted as follows: 0.01‐0.05 = *,0.01‐0.001 = **, 0.001‐0.0001 = *** *n* = 4.

To confirm the increase of lipids in EVs observed through RTM, we stained both EVs and parental hepatocytes from ZF and ZL rats with Bodipy, a highly lipophilic neutral fluorophore used to label a wide range of lipids (Elle et al., 2010; Karolin et al., 1994). Four hours after seeding the cells, it can be clearly appreciated that ZF primary hepatocytes accumulate in the cytoplasm larger amount of lipid droplets (LD) compared with ZL cells (Figure 2).

**FIGURE 3:**
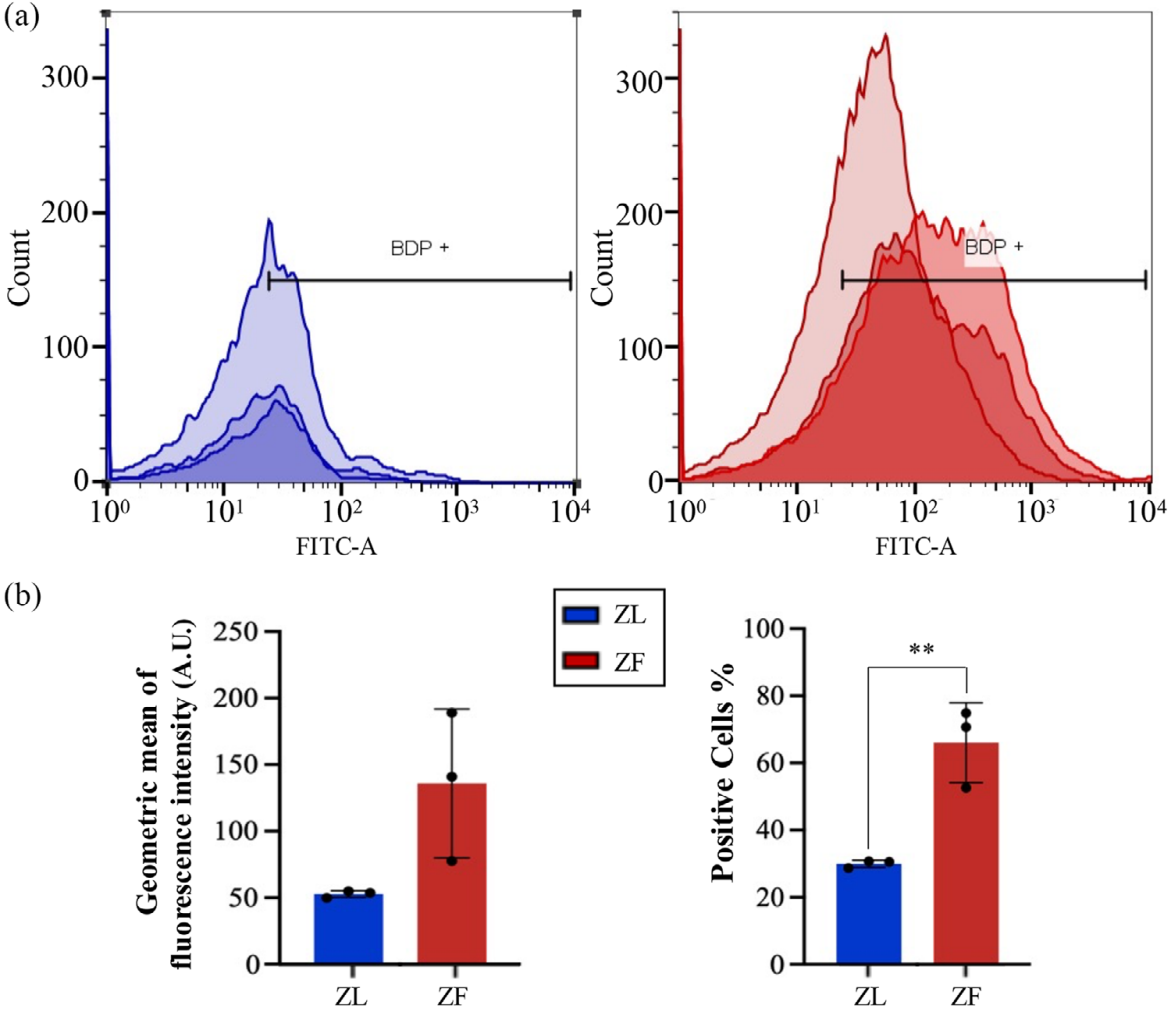
Direct Lipid content analysis in intact Zucker rat hepatocyte‐derived EVs. The content of lipids in EV samples was analysed by flow cytometry of Bodipy stained EV‐samples. (a) Overlay histograms of triplicates show the differences between ZL (blue) and ZF (red) EVs with (b) both the percentage of positive particles as well as the intensity means were increased in EVs derived from ZF hepatocytes, when compared with ZL EVs. Each biological replicate (*n* = 3) was calculated using 30,000 events.

This was also confirmed by a lipase assay, showing that ZF hepatocytes lysates treated with lipase released more glycerol, consistent with an increase of triglyceride content (Figure S4). Importantly, this increase of triglycerides was not due to changes in the abundance of lipoproteins as judged by the fact that the amount of Apolipoprotein B (ApoB) remains the same or increases in the ZL group (Figure S1B). As can be seen in Figure S1, unlike CD63, which exhibits a consistent pattern across the preparations, ApoB displays significant variability between samples. This observation reinforces the notion that the rise in triglycerides cannot be attributed to changes in ApoB. As it can be seen in Figure 2, although the ZF hepatocytes kept their integrity the differences showed at 4 h are not as evident after 36 h. Suggesting that they lose part of accumulated lipids, phenomena that could explain the higher proportion of lipids‐containing particles observed in RTM. In order to further validate these results, direct Bopidy staining of intact EVs was performed and analysed by flow cytometry (Figure 3a). The population of EVs was selected based on their forward and side scatter properties eliminating the background of PBS in which the EVs are resuspended (Figure S5A). Using the selected gate, the population of Bodipy positive particles (BDP+) were calculated by comparison with unstained EV preparation (Figures 3a and S5B). Three independent pairs of EVs were measured, and results are presented in Figure 3a as overlapping histograms of FITC‐A intensity. The results show an increase on both intensity and percentage of positive events in ZF versus ZL stained EVs (Figure 3b).

### 3.3 Lipidomic analysis

**FIGURE 4:**
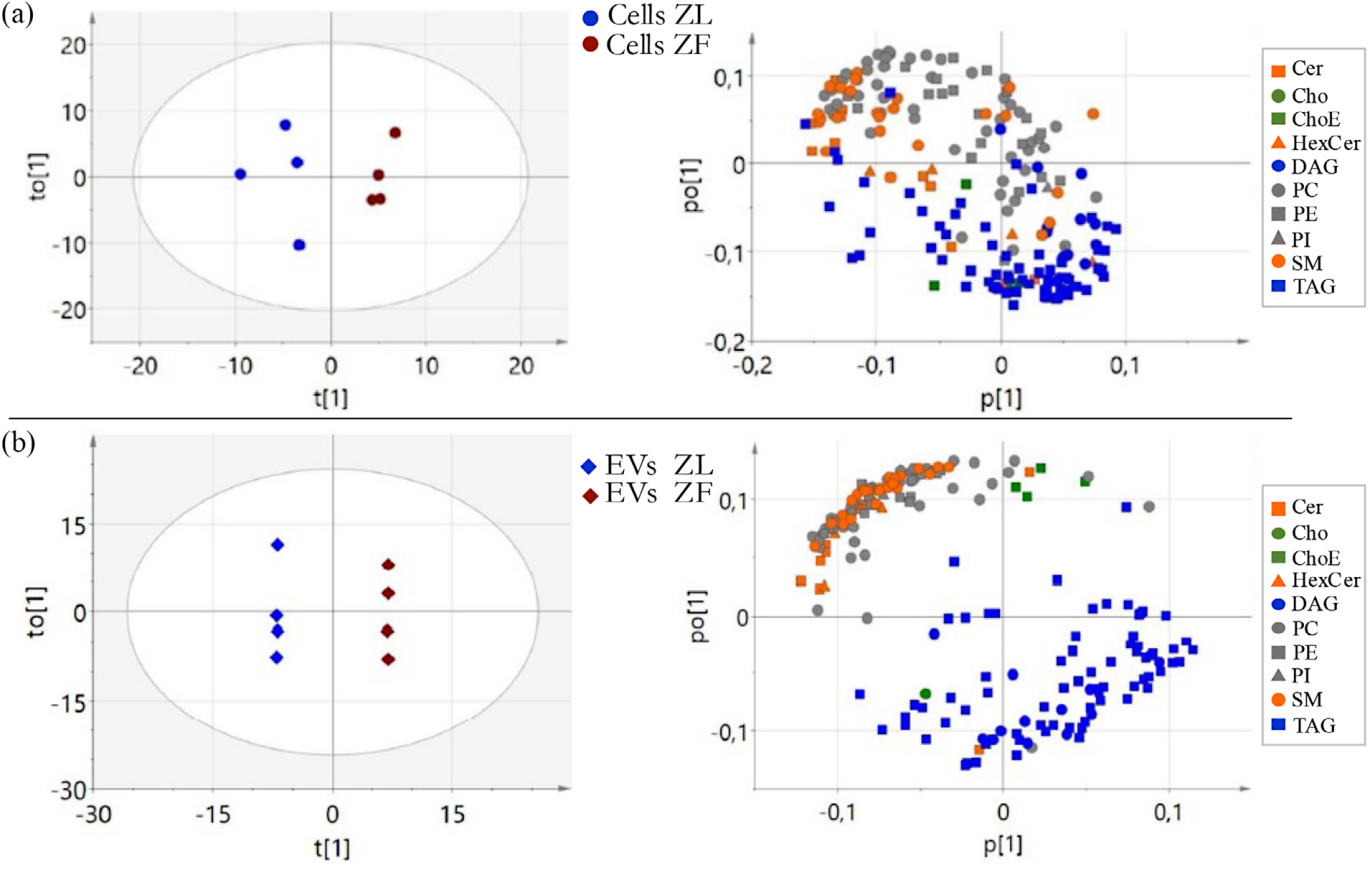
Lipidomic analysis. (a) Score scatter plot (left) and Loadings scatter plot (right) of the OPLS‐DA model of hepatocytes obtained from lean and obese rats. Model diagnostics (A = 3; R2X = 0.778; Q2X = 0.268). (b) Score plot (left) and loadings plot (right) of the OPLS‐DA model of EVs obtained from hepatocytes of ZL and ZF rats. Model diagnostics (A = 5; R2X = 0.904; Q2X = 0.976). 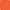 Cer: Ceramides; 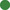 Cho: Cholesterol; 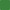 ChoE: Cholesteryl Esters; 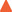
HexCer: Monohexosylceramides; 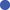 DAG: Diacylglycerols; 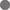 PC: Phosphatidylcholines; 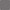 PE: Phosphatidylethanolamines; 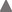
PI: Phosphoinositols; 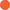 SM: Sphingolipids; 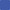 TAG: Triacylglycerols. n = 4 per condition.

Given the observed differences in lipid content between EVs derived from ZL and ZF rats, as well as in the corresponding primary hepatocytes cells, the next step was to determine which lipids were differently represented in the EV preparations and cells. A total of 199 metabolic features were obtained by a semitargeted UHPLC‐QTOF‐MS analysis, where glycerophospholipids, sterol lipids and sphingolipids were analysed and included in the subsequent univariate and multivariate data analysis. First, a visual inspection of the data by PCA analysis was performed to determine group tendencies (Figure S6). Clear separation between samples was not achieved but a tendency mainly through PC1 was found in the case of EVs by PCA (Figure S6). A supervised OPLS‐DA model was built in order to enhance group separation by adding class information. As it can be observed in Figure 4a, clear separation of ZF and ZL primary hepatocytes was achieved through OPLS‐DA model. Similarly, as seen in Figure 4b, EVs from ZL were perfectly separated from EVs ZL. Considering the overfitting nature of OPLS‐DA a proper model validation was required for variable selection. The CV‐ANOVA *p*‐value was calculated for both OPLS‐DA models. In both cases *p*‐values were greater than 0.05, 0.87 for cells (Figure 4a‐left) and 0.29 for EVs (Figure 4b‐left), which means not significant results (*p*‐value > 0.05). These lack of validation would be due to the small sample size and thus, variables could not be selected from these models. Despite this fact and taking into account the need of increasing the number of samples to obtain more robust results, loadings plots of both primary hepatocytes and their secreted EVs from OPLS‐DA models were further analysed to determine the contribution of different lipid classes. It was observed a similar increased tendency of glycerolipids (triacylglycerols, TAG; diacylglycerols, DAG) in cells and EVs from ZF rats compared to those obtained from ZL rats. On the contrary, glycerophospholipids (phosphatidylcholines, PC; phosphatidylethanolamines, PE; phosphatidylinositols, PI), and most ceramides (Cer) and sphingomyelins (SM), were increased in cells and EVs obtained from ZL rats compared to those obtained from ZF rats (see loadings plot of Figure 4a,b). Even though the lack of validation, this first approach of lipidomics to study differences in the lipid composition of EVs secreted from ZL and ZF hepatocytes open a new insight to further study the lipid content of these EVs.

Afterwards, a univariate analysis was performed using the student t‐test and the FDR approach. Among the 199 lipids, no significant (q‐value < 0.05) was obtained probably due to the number of samples in each group. However, the log2 fold‐change reveal very interesting results. The fold change as well as the statistical values of the 199 lipids are gathered in Table 1. Analysing all lipidomics findings of this pilot study, it can be pointed out the upregulated tendency of triacylglycerides in ZF primary hepatocytes when compared with ZL cells (see Table 1). This tendency is also translated into an enrichment on triacylglycerides in EV derived from ZF hepatocytes. Even though no significant results were obtained, comparing lipidomics results with other techniques included in this report several important findings were correlated. The upregulated tendency of triglycerides observed in ZF primary hepatocytes by lipidomics was on agreement with the increase of triglyceride content obtained by the lipase assay. This is of special interest due to two independent techniques resulted in same findings. This should be further study with an increased number of samples.

Regarding the rest of lipids classes, it was observed that most of sterols were upregulated in ZF cells and their corresponding EVs whereas glycerophospholipids were mainly downregulated in ZF cells and EVs. This last finding might be due to the imbalance of triacylglyceride species in ZF cells and EVs.

### 3.4 Biodistribution of ZF and ZL hepatocyte derived EVs

**FIGURE 5:**
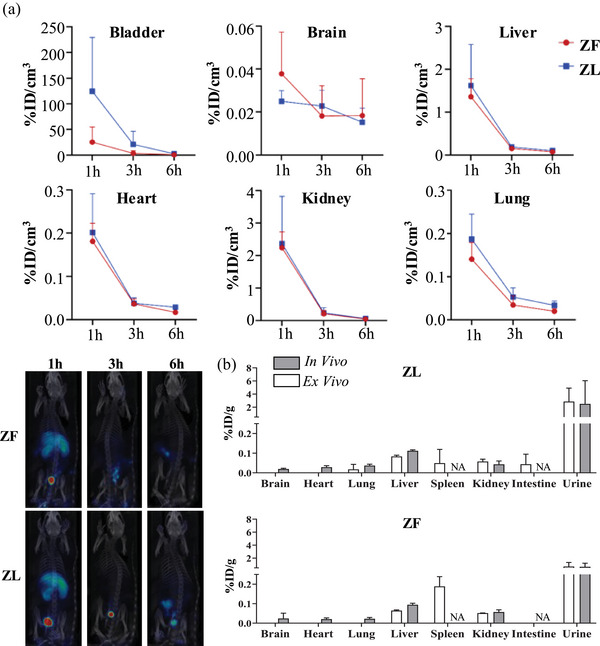
Biodistribution of Zucker rat hepatocyte‐derived EVs. (a) In vivo biodistribution of radiolabelled EVs from ZL and ZF rats, at t = 1, 3 and 6 h after intravenous administration, as determined by PET imaging. Left: representative PET images (maximum intensity projections, cor‐registered with rendered CT images; coronal view) for the different EVs at different time points; right; quantification values for the different organs. Values are expressed as mean ± standard deviation, *n* = 4 per EV and time point. (b) Concentration (%) of radioactivity in the different ex vivo organs at t = 6 h after intravenous administration of ZL and ZF EVs, as determined by dissection and gamma counting. Values are expressed as mean ± standard deviation, *n* = 4 per EV. NA: not applicable.

After the analysis of the composition of these EVs, we focused on the fate of these vesicles into the organism by comparing their biodistribution in rats. With that aim, EVs were radiolabelled with a pre‐labelled prosthetic group, [^18^F]F‐PyTFP, taking advantage of the free amino groups present on EVs. After incubation (15 min at 40°C) of EVs with [^18^F]F‐PyTFP, followed by a purification step by size exclusion chromatography, the radiochemical purity was ≥95%, as confirmed by radio‐TLC (Figure S7). Radiochemical yield was 42% and 45% (with respect to [^18^F]F‐PyTFP, decay corrected) for ZF and ZL EVs, respectively. Wistar control rats were injected via one of the lateral tail veins with radiolabelled EVs, and the accumulation in different organs was quantified using PET imaging at ca. 1 h, 3 and 6 h following administration (Figure 5a). At t = 1 h after administration, most of the radioactivity was found in the bladder, followed by the kidneys and the liver. Lower concentrations of radioactivity were measured in the lungs, the heart, and the brain. No significant differences were observed in the accumulation of ZF and ZL EVs in the different organs, except for the bladder at t = 6 h (*p* = 0.02). These differences disappeared at longer times post‐administration. In vivo results were then confirmed by *ex vivo* analysis. For this purpose, animals were sacrificed immediately after the last imaging session, and the concentration of radioactivity was determined using gamma counting. In this ex vivo experiment, two additional organs, the spleen and intestine, were analysed. The presence of surrounding organs obstructs signal detection in vivo, hence the necessity for ex vivo assessment. Equivalent results to those obtained from PET imaging were obtained in all organs except for the brain and the heart, where *ex vivo* studies revealed a negligible concentrations of radioactivity. The detection of radioactivity in these organs using PET can be attributed to the presence of blood (particularly in the heart) and the contribution of noise signals. Ex vivo studies revealed the presence of radioactivity in the spleen for both EVs, and in the small intestine for ZL EVs (Figure 5b).

## 4 DISCUSSION

Zucker rat is a model of obesity induced by a spontaneous mutation of the leptin receptor (Apweiler & Freund, 1993; Phillips et al., 1996). Carriers of this mutation develop obesity, hypercholesterolemia, insulin resistance and other symptoms related to metabolic syndrome, based on increased food consumption and reduced mobility. Primary hepatocytes obtained from this model show accumulation of LD in the cytoplasm just after cell seeding, but after some hours in culture, the lipid content is clearly reduced (Figure 2). Therefore, we hypothesize that lipids are released through EVs, supported by an increase in the lipid content of EV preparations derived from ZF hepatocytes (Figures 1 and 3). There is a great deal of controversy about the best method to isolate EVs for lipidomic studies, it has been shown that LDL and HDL can be copurified with EVs isolated by conventional ultracentrifugation, as they have similar biophysical properties (Sun et al., 2017, 2019). However, ultracentrifugation is a separation technique based on size and density. Therefore, EVs and VLDL would not be copurified since they have different densities (Liangsupree et al., 2021). Other works have reported an increase in the production of triglyceride‐rich lipoproteins in hepatocytes from ZF rats of the same age (ten weeks), for both triglyceride and ApoB (Chirieac et al., 2004). However, we did not observe an increment in the presence of ApoB in our EV preparations (Figure S1B), perhaps because ultracentrifugation samples are less prone to carry over low density lipoproteins (Azkargorta et al., 2021). Instead, we observed perilipin, a protein embedded in the monolayer of phospholipids and free cholesterol that surrounds LD (Ducharme & Bickel, 2008; Fujimoto et al., 2008). In fact, LDs are intracellular organelles specialized for the storage of TAG and cholesteryl ester in their core. It has been observed that adipocytes are able to release microvesicles (adiposomes) containing perilipin‐A together with other anchoring proteins of the LD, which may act upon neighbouring cells, preparing them for excessive TAG storage and LD biogenesis (Müller et al., 2009). The release of TAG inside vesicles has not been probed, even in EVs isolated from an environment rich in TAG like milk (Blans et al., 2017), and therefore the risk of contamination through LD or lipoproteins (Skotland et al., 2020) could also be the case in our preparations, given that we detected a low level of ApoB. Despite detecting ApoB in our samples, no significant differences were detected in lipoprotein content, suggesting no differences in lean and fatty hepatocyte derived‐EVs.

Our group already reported, through lipidomic profiling, the abundance of TAG in EV preparations coming from primary rat hepatocytes (Royo, Gil‐Carton et al., 2019). Although we could not probe the presence of TAG within double membrane vesicles, we had observed a shift from monoacyl to diacylglycerophosphocholines that will have an impact on the asymmetry of the membranes, favouring the transport of certain lipids (Weijers, 2012). In the present analysis, we observed TAG in both samples, with a clear enrichment in ZF compared to ZL hepatocytes (Table 1). Blandin et al. in a study in which they analysed lipid content of adipocytes derived‐EVs from obese and lean mice, they found that those EVs contained detectable amounts of TAG. In this case, no clear difference in TAG was seen between lean and obese samples, but an increase in TAG was seen when analysing visceral adipose tissue (Blandin et al., 2023). In addition, in this study they found enrichment of Cer, SM, and phosphatidylglycerols (PE, PC, PI) species in adipocytes derived EVs from lean and obese mice compared to visceral adipose tissue, a phenomenon we have seen in EVs derived from ZL hepatocytes. Moreover, patient‐derived adipose EVs were found to impact cancer development, likely due to specific modifications of lipidic composition in the tumour environment, RNA, and protein content that may act synergistically (Le Lay et al., 2021). Analogously, we can hypothesize that the observed changes in lipidic composition between ZF and ZL hepatocytes and EVs may have an impact in those areas where liver derived EVs accumulate, for instance in the hypothalamus, as we had observed in our study.

However, in spite of being enriched in cells, species with a chain with odd of carbons are less enriched in ZF EVs, that is, TG 47:1, TG47:2, TG49:2, TG55:1, TG55:2, TG55:4. This selectivity on the enrichment of TAG in the preparations suggests a difference in the packaging of lipids upon release, where certain species can be released through a new way, likely involving perilipin, while other species are released through a similar packaging that we already observed in EVs released by primary hepatocytes of healthy Wistar rats (Royo, Gil‐Carton et al., 2019). This would agree with the classification of particles performed with Raman spectroscopy, where the main differences are observed mainly in very lipidic particles (that may be floating LD, or protein‐saturated lipid particles, compatible with apolipoproteins) (Figure 1). Finally, the increase in TAG species correlates with underrepresentation of species in other lipid families, named phosphatidylcholines, phosphatidylethanolamines, phosphatidylinositols, and most ceramides and sphingomyelins.

Knowing that EV preparations were enriched with a differential load of lipids, the next question was to determine whether EVs derived from hepatocytes had any effect on other organs. We have previously observed circulating EVs carrying proteins from the liver (Palomo et al., 2018; Palomo et al., 2014). In the biodistribution assay (Figure 5a), we detected EVs in liver, kidney and bladder, and to a lower extent in the brain, heart, and lungs after 1 h from injection. However, after 6 h from administration, the majority of the EVs were eliminated across urine. Although these results do not accurately provide us with the physiological biodistribution of vesicles, this experiment brings us closer to understanding their physiological significance compared to in vitro EV functional studies (Kang et al., 2021). Wiklander et al. studied the biodistribution of EVs from different species origins and showed same pattern of biodistribution for EVs derived from mouse, rat and human cell lines. Besides, they found that the administration route and the injected dose could influence the biodistribution pattern (Wiklander et al., 2015). To complement the information acquired through the In vivo experiment, we also measured *ex vivo* the presence of radioactivity in different organs after 6 h of injection (Figure 5b). As previously seen in other studies, most of the signal was detected in the liver and spleen (Morishita et al., 2015; Németh et al., 2021; Takahashi et al., 2013). Brain, lung and kidney had equal expression in vivo and ex vivo. On the contrary, signals in the heart and brain were not detectable *ex vivo*.

Nevertheless, we and others have observed in vivo and ex vivo the presence of labelled EVs, which had certain ability to pass through the blood brain barrier (Royo, Cossío et al., 2019; Schiera et al., 2015). Considering that EVs load TAGs, it is possible to hypothesize that EVs may had an impact on nutritional behaviour, since it has been found that medium‐chain TAGs are the once with most potential to enhance satiety (Hamad et al., 2020; Maher & Clegg, 2019).

## 5 CONCLUSION

Although further studies are required to enhance the robustness and the findings obtained in this pilot study by increasing the number of samples, the result here presented are on agreement with other scientific reports and demonstrated the existence of differences between ZF and ZL cells and EVs. Furthermore, the fact that EVs released by a fatty liver have different lipid signature, together with the capability of EVs of reaching different organs, opens a new insight for both pathology studies and therapeutic interventions.

## AUTHOR CONTRIBUTIONS

Conceptualization, M.A.A., F.R., J.M., S.K., J.L, C.S., C.A. and J.M.F.P.; Data Curation, M.I. and J.K.; Formal Analysis, J.M., O.E.A., S.K., J.L, C.S., C.A. and J.M.G.; Funding Acquisition, J.M.F.P., Investigation, M.A.A., F.R., J.M., S.K., J.L, C.S. and C.A.; Methodology, M.A.A., F.R., J.M., S.K., J.L, C.S., C.A. E.G., C.G.V., M.I. and J.M.F.P.; Project Administration, J.M.F.P.;, Resources, S.K., C.A., J.L. and J.M.P.F.; Software, S.K., C.S., C.A. and J.M.G.; Supervision, J.M.F.P.; Validation, M.A.A., F.R., J.M., S.K., J.L, C.S., C.A. and J.M.F.P.; Visualization, M.A.A.; Writing—Original Draft Preparation, M.A.A., F.R., J.M., S.K., J.L., C.A. and J.M.F.P.; Writing—Review & Editing, M.A.A., O.E.A., F.R., J.M., S.K., J.L, C.S., C.A. and J.M.F.P.

## CONFLICT OF INTEREST STATEMENT

The authors declare no conflict of interest.

## Supporting information

Supplementary Information

Supplementary Information

Supplementary Information

Supplementary Information

Supplementary Information

Supplementary Information

Supplementary Information

Supplementary Information

Supplementary Information

Supplementary Information

## Data Availability

Data will be made freely available if request.
