# Supplementary material for: Lipidomics and biodistribution of extracellular vesicles‐secreted by hepatocytes from Zucker lean and fatty rats
Source: J Extracell Biol. 2024 Feb 22;3(2):e140. doi: 10.1002/jex2.140 (PMC11080883; doi:10.1002/jex2.140)
Supplement: Supplementary file 1 — Supplementary Information [file JEX2-3-e140-s001.docx]

| **Antibody** | **Species** | **Clone** | **ID** | **Supplier** | **Dilution** |
| --- | --- | --- | --- | --- | --- |
| **AIP1** | Mouse | 49 | 554002 | BD | 1:1000 |
| **ApoB (48/100)** | Rabbit |  | BP2050 | Origene | 1:1000 |
| **CD63** | Mouse | AD1 | ab108950 | Abcam | 1:1000 |
| **COXIV** | Rabbit | 3E11 | 4850 | Cell Signaling | 1:1000 |
| **Perilipin** | Rabbit |  |  |  | 1:1000 |

**Table S1: List of antibodies**
