# Supplementary material for: Lipidomics and biodistribution of extracellular vesicles‐secreted by hepatocytes from Zucker lean and fatty rats
Source: J Extracell Biol. 2024 Feb 22;3(2):e140. doi: 10.1002/jex2.140 (PMC11080883; doi:10.1002/jex2.140)
Supplement: Supplementary file 2 — Supplementary Information [file JEX2-3-e140-s005.docx]

**Table S2: UHPLC-MS analysis methods**. Chromatographic separation and mass spectrometric detection conditions.

| **Column type** | UPLC BEH C18, 1.0 x 100 mm, 1.7 μm |
| --- | --- |
| **Flow rate** | 0.15 ml/min |
| **Solvent A** | H2O + ACN + 10mM Ammonium Formate |
| **Solvent B** | ACN+ Isopropanol + 10mM Ammonium Formate |
| **(%B), time** | 40%, 0 min |
| **(%B), time** | 100%, 10 min |
| **(%B), time** | 40%, 15 min |
| **(%B), time** | 40%, 17 min |
| **Column temperature** | 60 °C |
| **Injection volume** | 2 μl |
| **Source temperature** | 120 °C |
| **Nebulisation N_2_ flow** | 1000 l / hour |
| **Nebulisation N_2_ temperature** | 400 °C |
| **Cone N_2_ flow** | 30 l / hour |
| **Capillary voltage** | 2 kV |
| **Cone voltage** | 30 V |
