# Supplementary material for: Lipidomics and biodistribution of extracellular vesicles‐secreted by hepatocytes from Zucker lean and fatty rats
Source: J Extracell Biol. 2024 Feb 22;3(2):e140. doi: 10.1002/jex2.140 (PMC11080883; doi:10.1002/jex2.140)
Supplement: Supplementary file 3 — Supplementary Information [file JEX2-3-e140-s004.pdf]

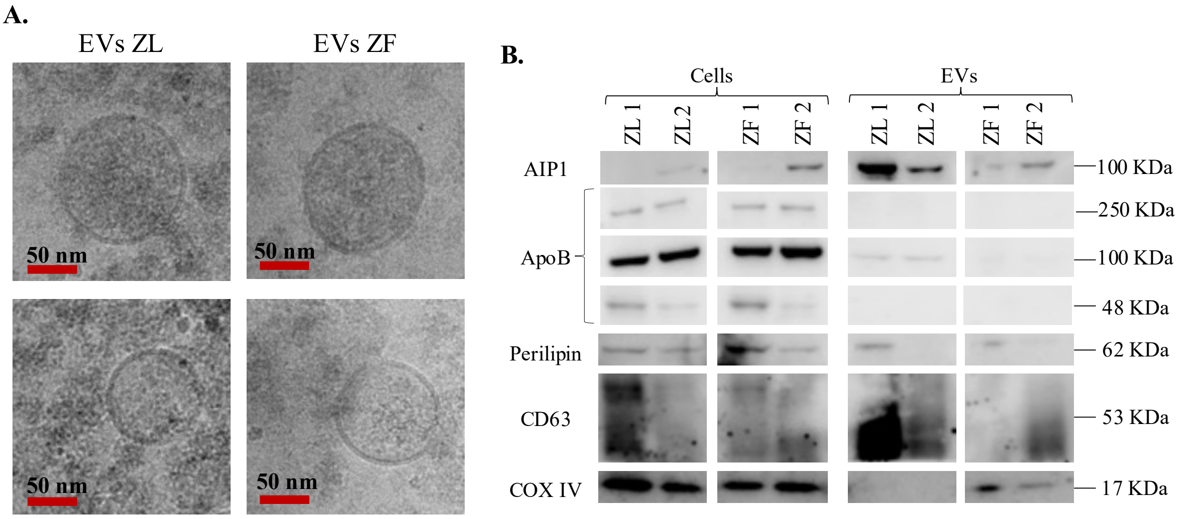

Figure S1: Characterization of EVs obtained after isolation by ultracentrifugation. (A) Characterization of EVs by Cryo-EM. **(B)** Protein characterization by Western blotting by using antibodies against the indicated proteins (20µg) n=3.
