# Supplementary figures and images for: Lipidomics and biodistribution of extracellular vesicles‐secreted by hepatocytes from Zucker lean and fatty rats
Source: J Extracell Biol. 2024 Feb 22;3(2):e140. doi: 10.1002/jex2.140 (PMC11080883; doi:10.1002/jex2.140)

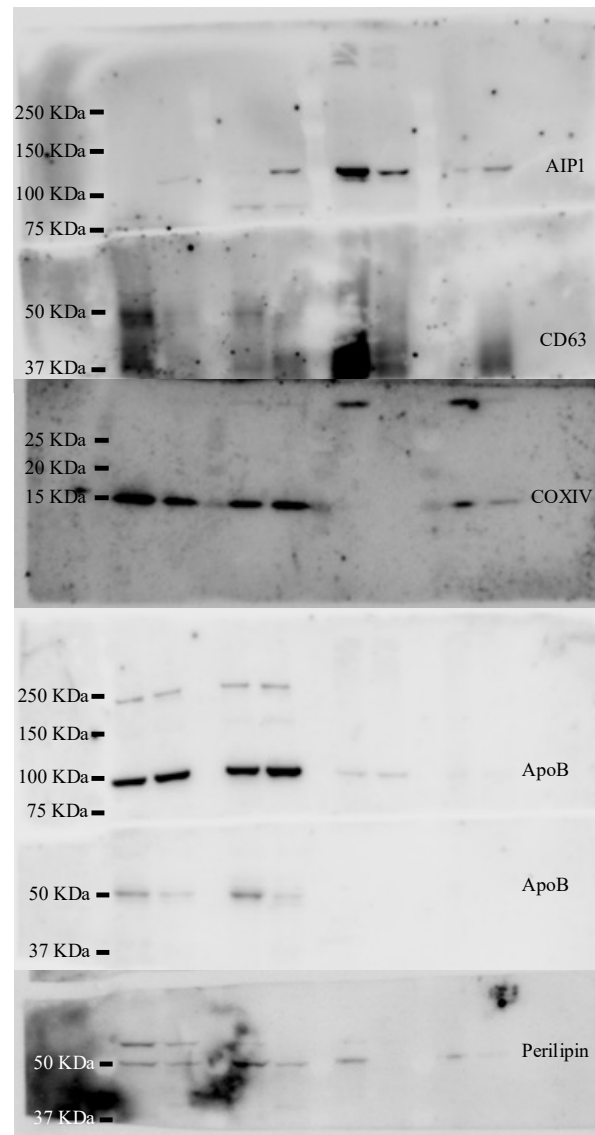

**Figure S2: Entire electrophoretogram of Figure S1B.**

Supplement: Supplementary file 4 — Supplementary Information [file JEX2-3-e140-s009.pdf]
