# Supplementary material for: Lipidomics and biodistribution of extracellular vesicles‐secreted by hepatocytes from Zucker lean and fatty rats
Source: J Extracell Biol. 2024 Feb 22;3(2):e140. doi: 10.1002/jex2.140 (PMC11080883; doi:10.1002/jex2.140)
Supplement: Supplementary file 5 — Supplementary Information [file JEX2-3-e140-s007.pdf]

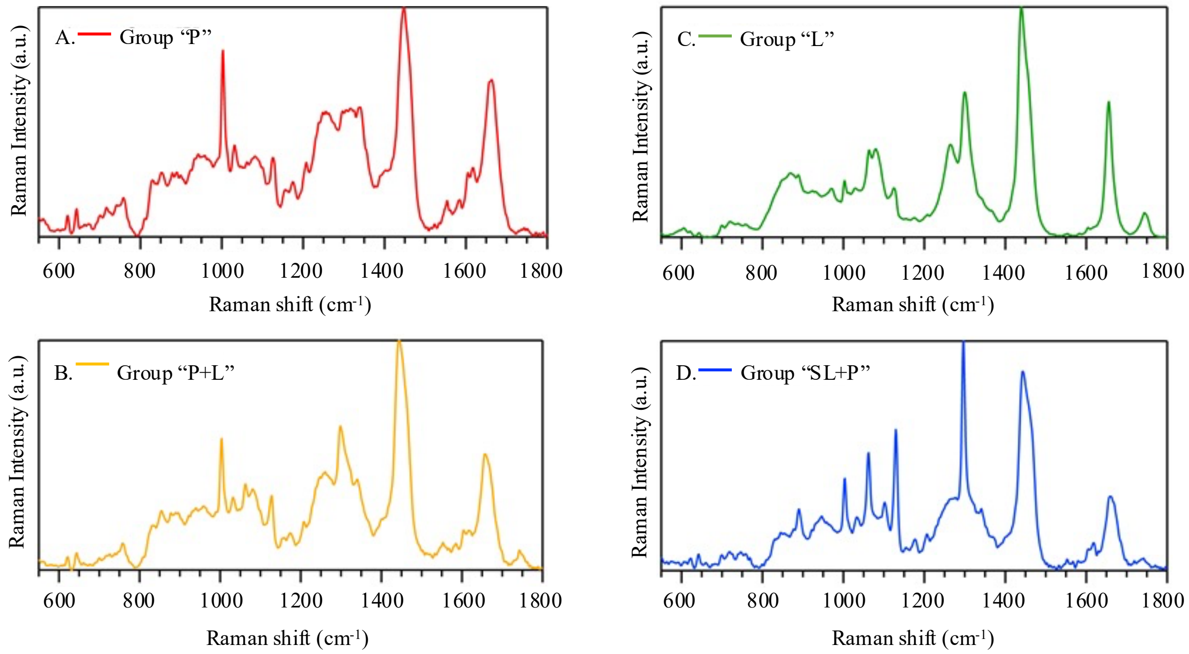

Figure S3: Raman spectra. Typical characteristic Raman spectra of four different biomolecular groups found in our small EVs preparations; these spectra were used as a reference for recorded Raman spectra attribution. Biomolecular group “P” dominant protein contribution, with very weak or negligible lipid content. Group “P+L” proteins and lipids in various non-negligible proportions. Group “L” dominant unsaturated lipids, negligible proteins; Group “SL+P” saturated lipids and proteins in various proportions, together with variable proteins content. This last group was specifically defined to evaluate the major contribution from saturated lipids.
