# Supplementary material for: Lipidomics and biodistribution of extracellular vesicles‐secreted by hepatocytes from Zucker lean and fatty rats
Source: J Extracell Biol. 2024 Feb 22;3(2):e140. doi: 10.1002/jex2.140 (PMC11080883; doi:10.1002/jex2.140)
Supplement: Supplementary file 6 — Supplementary Information [file JEX2-3-e140-s008.pdf]

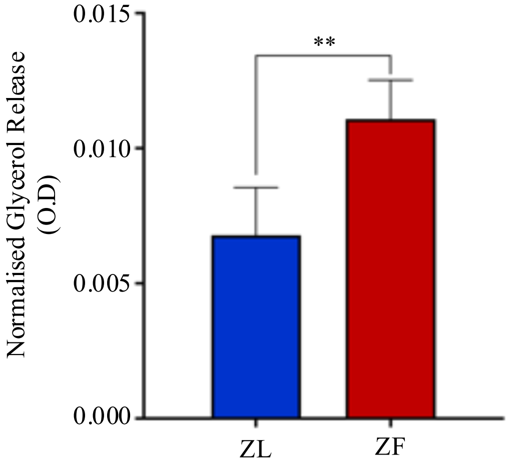

Figure S4. Triglyceride content in Zucker rat hepatocytes. Triglyceride content of primary Zucker rat hepatocytes was measured using lipase activity assay. The p values were denoted as follows: 0.01-0.05=*,0.01-0.001=**, 0.001-0.0001=***n=4.
