# Supplementary material for: Lipidomics and biodistribution of extracellular vesicles‐secreted by hepatocytes from Zucker lean and fatty rats
Source: J Extracell Biol. 2024 Feb 22;3(2):e140. doi: 10.1002/jex2.140 (PMC11080883; doi:10.1002/jex2.140)
Supplement: Supplementary file 7 — Supplementary Information [file JEX2-3-e140-s003.pdf]

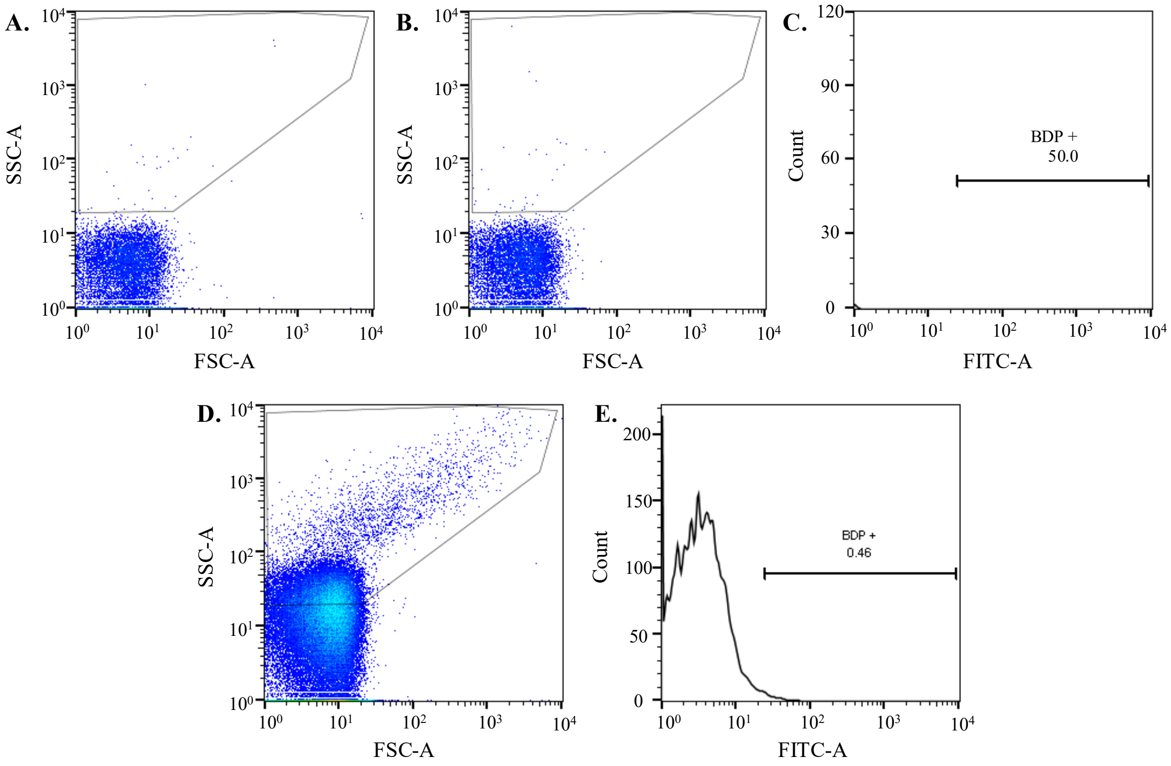

Figure S5: Flow cytometry setting analysis of EV preparations. (A) Cells stained with PBS to define the gate of cell derived particles**. (B)** Bodipy staining control for discard **(C)** unspecific signal of the fluorocrome. The EV preparations were analysed with the same gate.**(D)** Non-stained EVs were employed to define **(E)** the Bodipy positive population (BDP+).
