# Supplementary material for: Lipidomics and biodistribution of extracellular vesicles‐secreted by hepatocytes from Zucker lean and fatty rats
Source: J Extracell Biol. 2024 Feb 22;3(2):e140. doi: 10.1002/jex2.140 (PMC11080883; doi:10.1002/jex2.140)
Supplement: Supplementary file 8 — Supplementary Information [file JEX2-3-e140-s010.pdf]

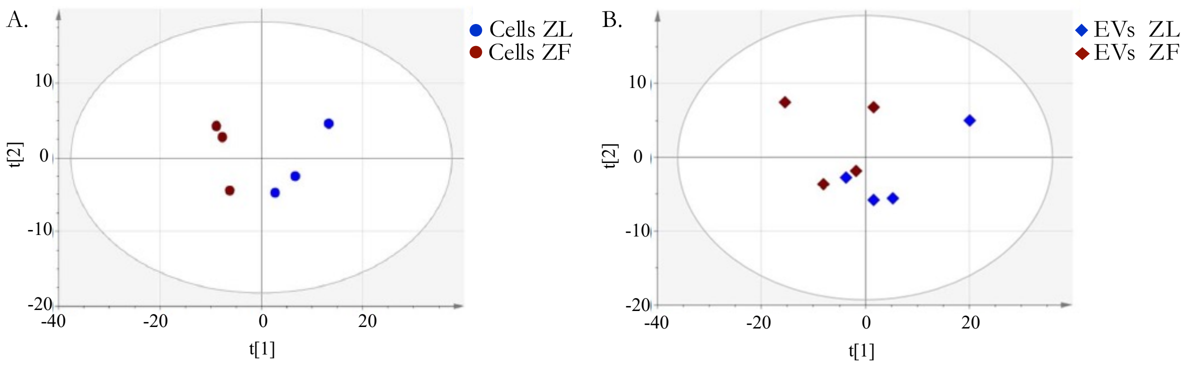

Figure S6: Score scatter plot of (A) PCA model of cells obtained from lean and obese rats**.** Model diagnostics (A=3; R2X= 0.874; Q2X=0.609)**. (B)** PCA model of EVs obtained from ZL and ZF rats. Model diagnostics (A=2; R2X=0.701; Q2X=0.302)
