# Supplementary material for: Lipidomics and biodistribution of extracellular vesicles‐secreted by hepatocytes from Zucker lean and fatty rats
Source: J Extracell Biol. 2024 Feb 22;3(2):e140. doi: 10.1002/jex2.140 (PMC11080883; doi:10.1002/jex2.140)
Supplement: Supplementary file 9 — Supplementary Information [file JEX2-3-e140-s006.pdf]

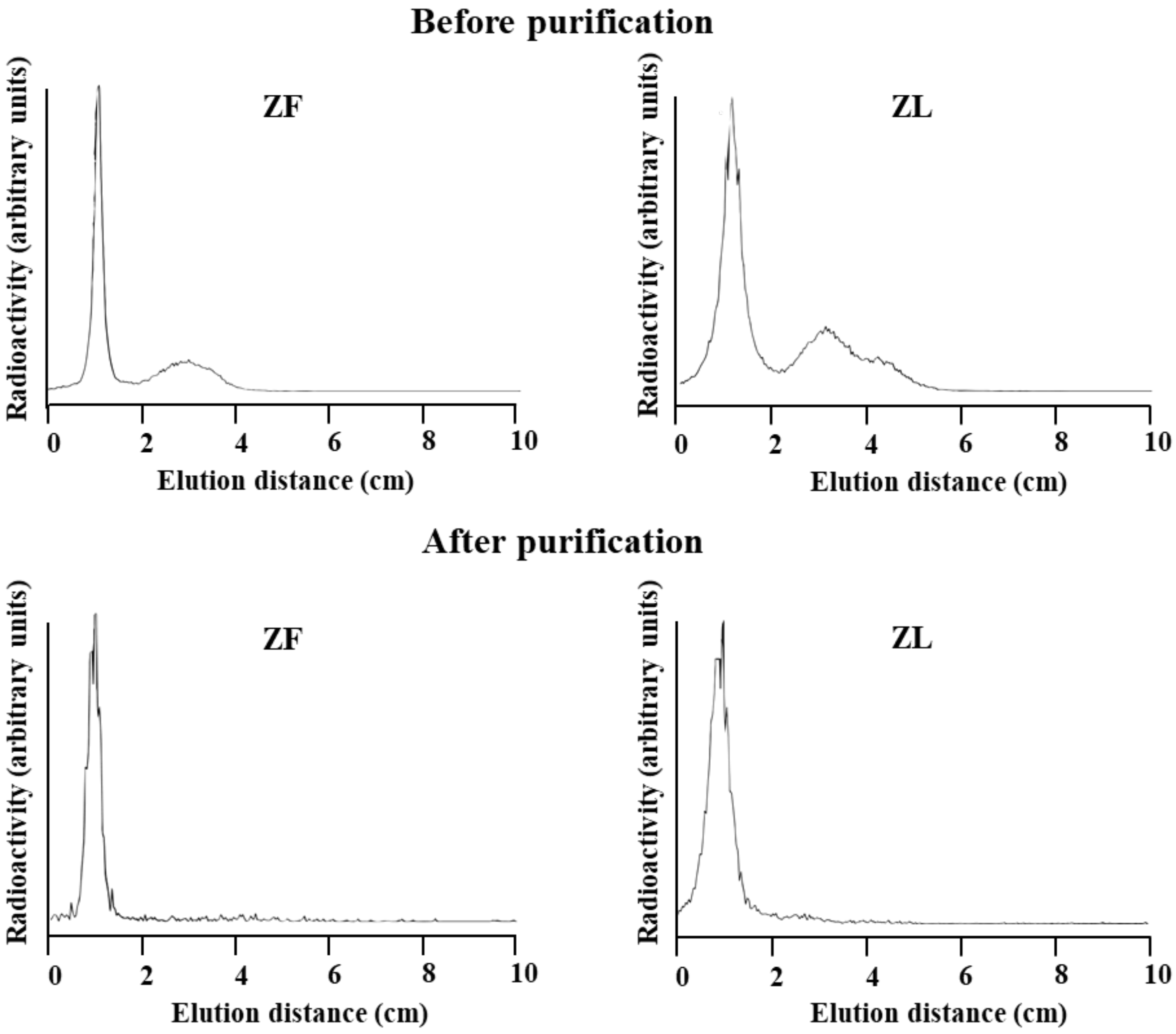

Figure S7: Representative radioactive chromatograms obtained for ZF and ZL EVs before (top) and after (bottom) purification by size exclusion chromatography
