# Supplementary material for: Lipidomics and biodistribution of extracellular vesicles‐secreted by hepatocytes from Zucker lean and fatty rats
Source: J Extracell Biol. 2024 Feb 22;3(2):e140. doi: 10.1002/jex2.140 (PMC11080883; doi:10.1002/jex2.140)
Supplement: Supplementary file 10 — Supplementary Information [file JEX2-3-e140-s002.docx]

**Supplementary material**

**Table S1: List of antibodies**

| **Antibody** | **Species** | **Clone** | **ID** | **Supplier** | **Dilution** |
| --- | --- | --- | --- | --- | --- |
| **AIP1** | Mouse | 49 | 554002 | BD | 1:1000 |
| **ApoB (48/100)** | Rabbit |  | BP2050 | Origene | 1:1000 |
| **CD63** | Mouse | AD1 | ab108950 | Abcam | 1:1000 |
| **COXIV** | Rabbit | 3E11 | 4850 | Cell Signaling | 1:1000 |
| **Perilipin** | Rabbit |  |  |  | 1:1000 |

| **Column type** | UPLC BEH C18, 1.0 x 100 mm, 1.7 μm |
| --- | --- |
| **Flow rate** | 0.15 ml/min |
| **Solvent A** | H2O + ACN + 10mM Ammonium Formate |
| **Solvent B** | ACN+ Isopropanol + 10mM Ammonium Formate |
| **(%B), time** | 40%, 0 min |
| **(%B), time** | 100%, 10 min |
| **(%B), time** | 40%, 15 min |
| **(%B), time** | 40%, 17 min |
| **Column temperature** | 60 °C |
| **Injection volume** | 2 μl |
| **Source temperature** | 120 °C |
| **Nebulisation N_2_ flow** | 1000 l / hour |
| **Nebulisation N_2_ temperature** | 400 °C |
| **Cone N_2_ flow** | 30 l / hour |
| **Capillary voltage** | 2 kV |
| **Cone voltage** | 30 V |

**Table S2: UHPLC-MS analysis methods**. Chromatographic separation and mass spectrometric detection conditions.

**
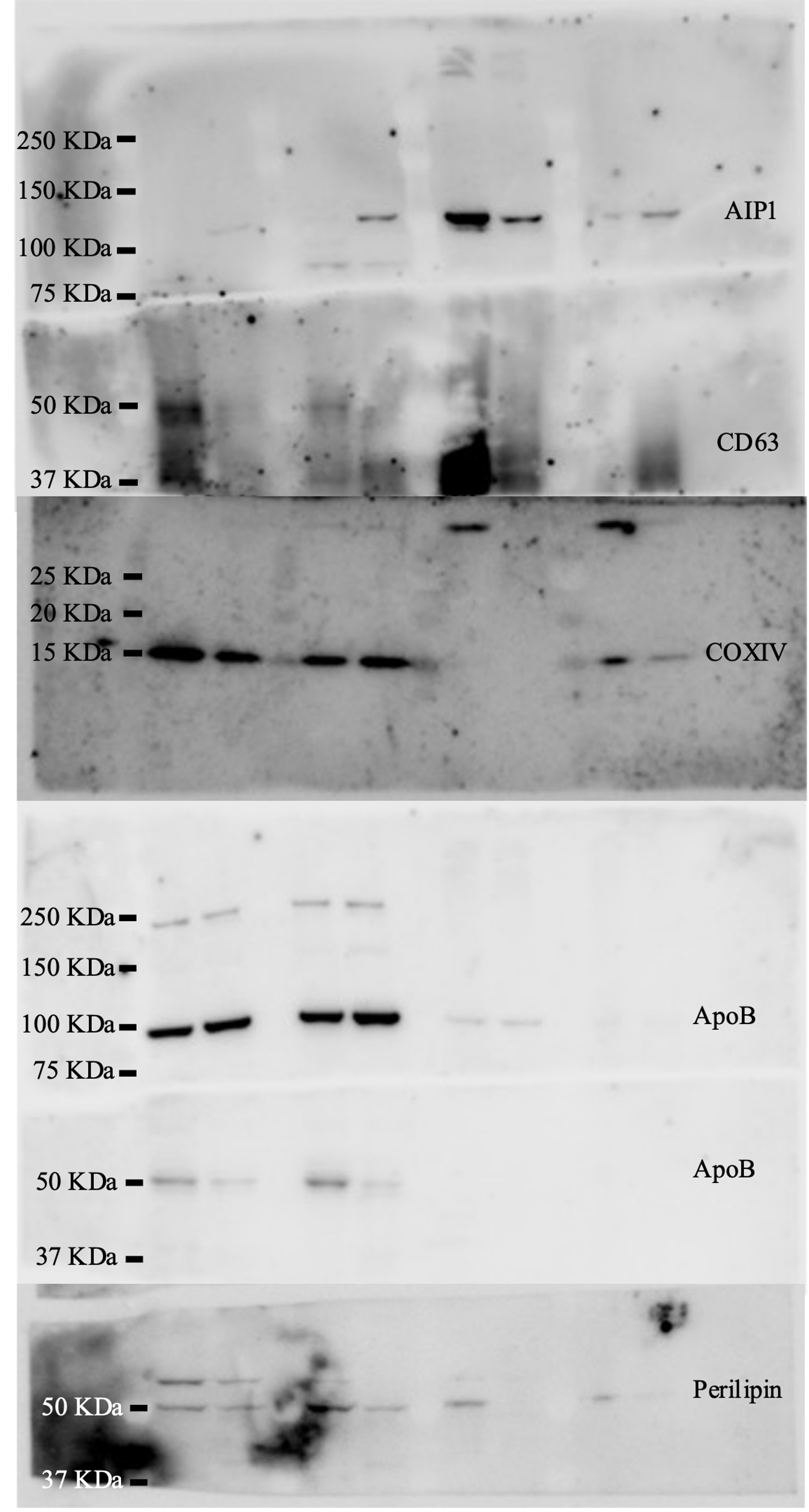
** **
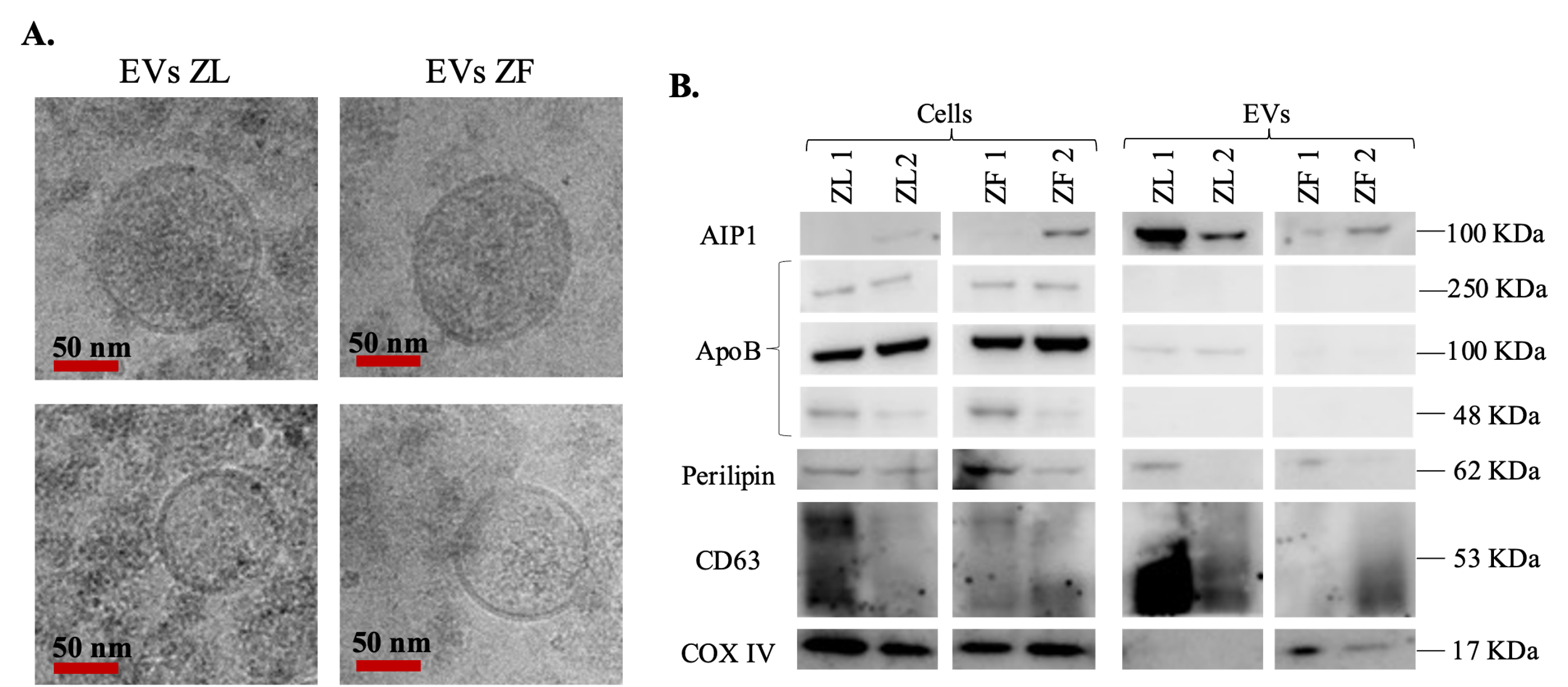
**

**Figure S1: Characterization of EVs obtained after isolation by ultracentrifugation.** **(A)** Characterization of EVs by Cryo-EM. **(B)** Protein characterization by Western blotting by using antibodies against the indicated proteins (20μg) n=3.

**Figure S2: Entire electrophoretogram of Figure S1B.**

**
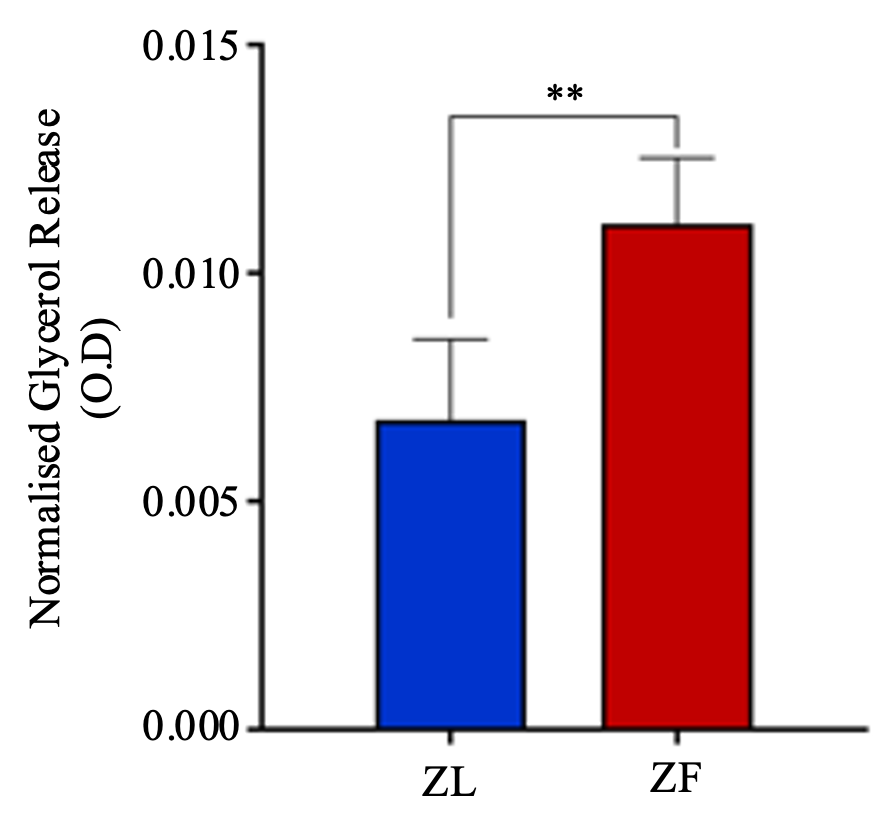

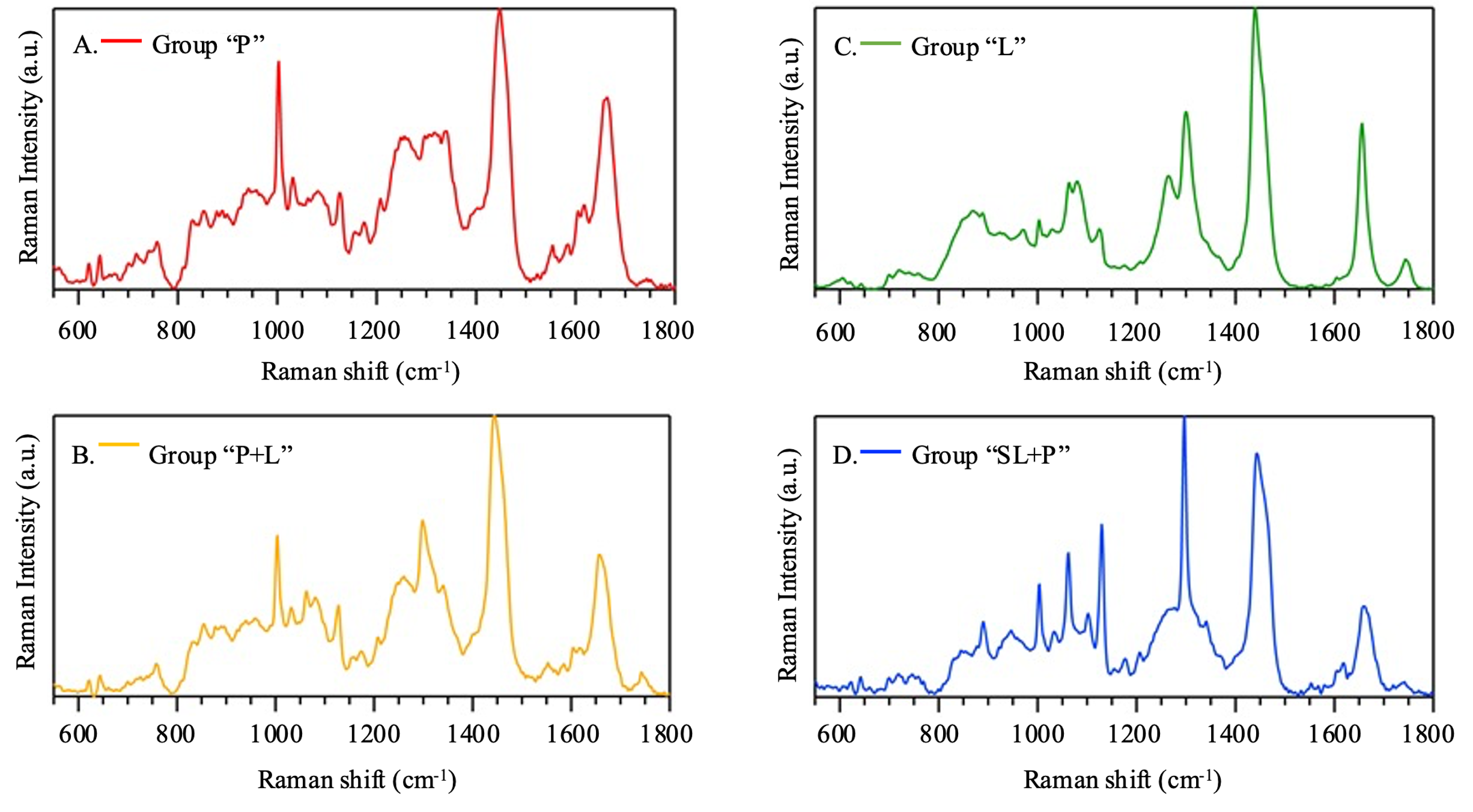
**

**Figure S3: Raman spectra.** Typical characteristic Raman spectra of four different biomolecular groups found in our small EVs preparations; these spectra were used as a reference for recorded Raman spectra attribution. Biomolecular group “P” dominant protein contribution, with very weak or negligible lipid content. Group “P+L” proteins and lipids in various non-negligible proportions. Group “L” dominant unsaturated lipids, negligible proteins; Group “SL+P” saturated lipids and proteins in various proportions, together with variable proteins content. This last group was specifically defined to evaluate the major contribution from saturated lipids.

**Figure S4. Triglyceride content in Zucker rat hepatocytes**. Triglyceride content of primary Zucker rat hepatocytes was measured using lipase activity assay. The p values were denoted as follows: 0.01-0.05=*,0.01-0.001=**, 0.001-0.0001=***n=4.

**
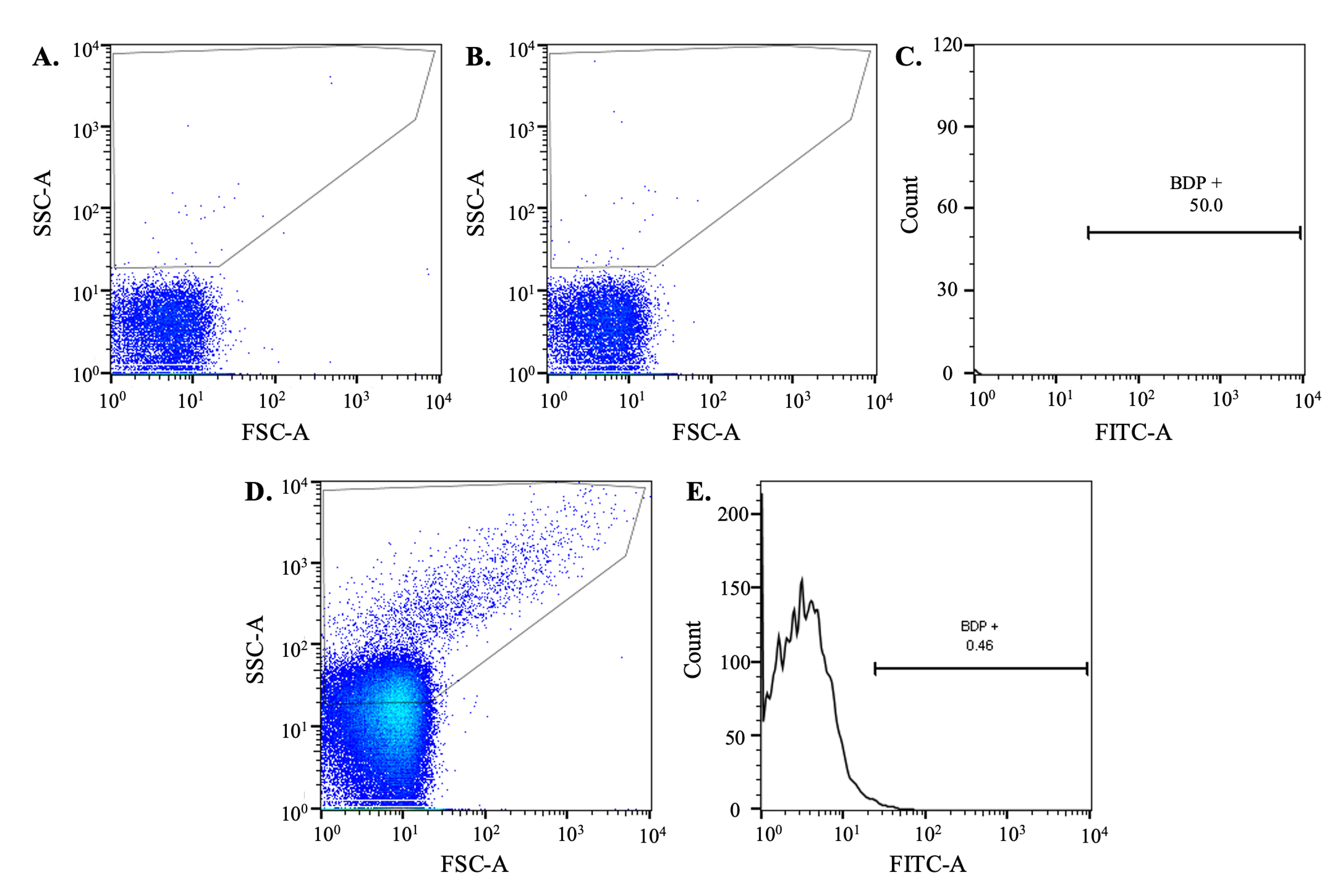
**

**Figure S5:** **Flow cytometry setting analysis of EV preparations**. **(**A) Cells stained with PBS to define the gate of cell derived particles**. (B)** Bodipy staining control for discard **(C)** unspecific signal of the fluorocrome. The EV preparations were analysed with the same gate.**(D)** Non-stained EVs were employed to define **(E)** the Bodipy positive population (BDP+).

**Figure S6: Score scatter plot of (A)** PCA model of cells obtained from lean and obese rats**.** Model diagnostics (A=3; R2X= 0.874; Q2X=0.609)**. (B)**  PCA model of EVs obtained from ZL and ZF rats. Model diagnostics (A=2; R2X=0.701; Q2X=0.302)

**
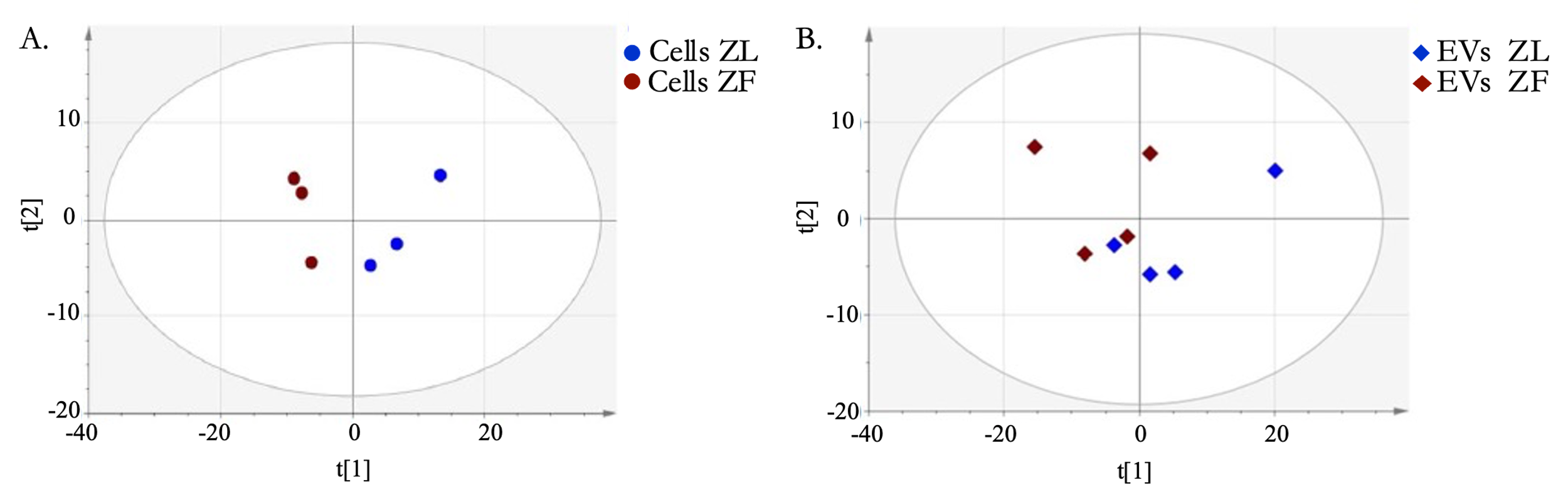
**

**
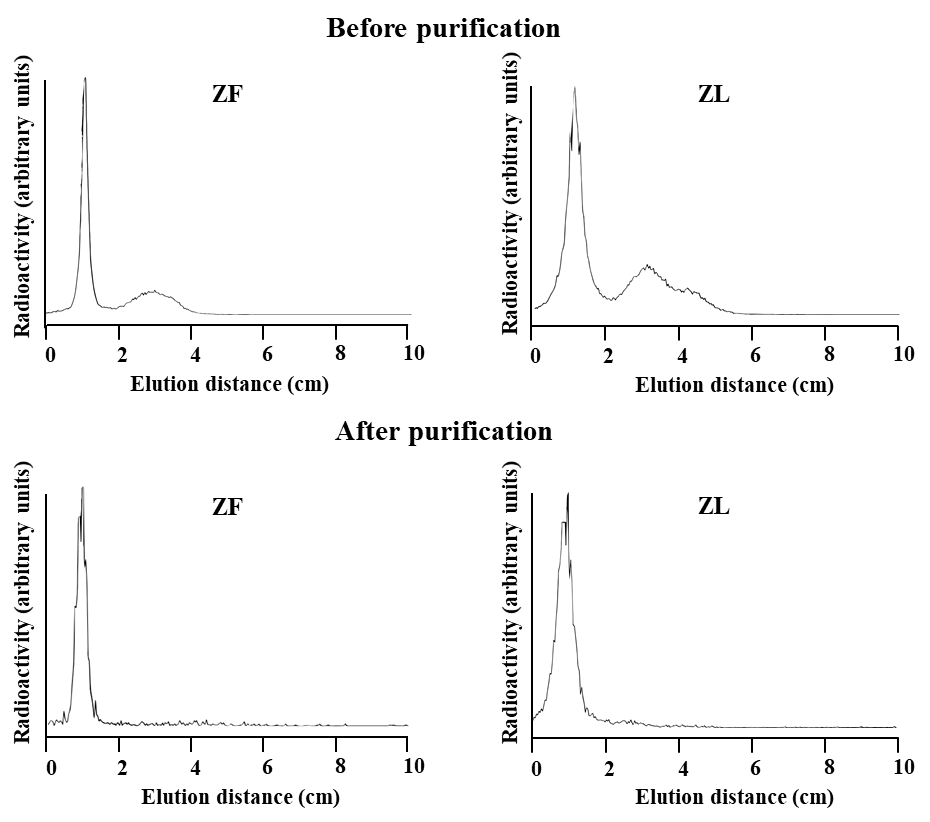
**

**Figure S7: Representative radioactive chromatograms** obtained for ZF and ZL EVs before (top) and after (bottom) purification by size exclusion chromatography
